# The *Wolfiporia cocos* Genome and Transcriptome Shed Light on the Formation of Its Edible and Medicinal Sclerotium

**DOI:** 10.1016/j.gpb.2019.01.007

**Published:** 2020-12-24

**Authors:** Hongmei Luo, Jun Qian, Zhichao Xu, Wanjing Liu, Lei Xu, Ying Li, Jiang Xu, Jianhong Zhang, Xiaolan Xu, Chang Liu, Liu He, Jianqin Li, Chao Sun, Francis Martin, Jingyuan Song, Shilin Chen

**Affiliations:** 1 Engineering Research Center of Chinese Medicine Resource, Ministry of Education, Institute of Medicinal Plant Development, Chinese Academy of Medical Sciences & Peking Union Medical College, Beijing 100193, China; 2 College of Pharmacy, Hubei University of Chinese Medicine, Wuhan 430065, China; 3 Institute of Chinese Materia Medica, China Academy of Chinese Medical Sciences, Beijing 100700, China; 4 INRA, Université de Lorraine, UMR 1136 Interactions Arbres/Microorganismes, 54280 Champenoux, France; 5 Beijing Advanced Innovation Center for Tree Breeding by Molecular Design, Institute of Microbiology, Beijing Forestry University, Beijing 100083, China

**Keywords:** *Wolfiporia cocos*, Genome sequencing, Sclerotial formation, Fungal development, Polysaccharide and triterpenoid biosynthesis

## Abstract

***Wolfiporia cocos*** (F. A. Wolf) has been praised as a food delicacy and medicine for centuries in China. Here, we present the genome and transcriptome of the Chinese strain CGMCC5.78 of *W. cocos*. High-confidence functional prediction was made for 9277 genes among the 10,908 total predicted gene models in the *W. cocos* genome. Up to 2838 differentially expressed genes (DEGs) were identified to be related to sclerotial development by comparing the transcriptomes of mycelial and sclerotial tissues. These DEGs are involved in mating processes, differentiation of fruiting body tissues, and metabolic pathways*.* A number of genes encoding enzymes and regulatory factors related to polysaccharide and triterpenoid production were strikingly regulated*.* A potential triterpenoid gene cluster including the signature lanosterol synthase (*LSS*) gene and its modified components were annotated. In addition, five nonribosomal peptide synthase (*NRPS*)-like gene clusters, eight polyketide synthase (*PKS*) gene clusters, and 15 terpene gene clusters were discovered in the genome. The differential expression of the velevt family proteins, transcription factors, carbohydrate-active enzymes, and signaling components indicated their essential roles in the regulation of **fungal development** and secondary metabolism in *W. cocos.* These genomic and transcriptomic resources will be valuable for further investigations of the molecular mechanisms controlling **sclerotial formation** and for its improved medicinal applications.

## Introduction

*Wolfiporia cocos* (F. A. Wolf) Ryvarden & Gilb., a member of the Polyporaceae family, is one of the medicinal mushrooms forming edible sclerotium. *W. cocos* is also a well-known brown rot species widely distributed around the world [1], [2]. As one of the Aphyllophorales fungi, *W. cocos* parasitizes the roots of conifers (*e.g.*, *Picea*, *Tsuga*, and *Pinus*) and hardwood trees (*e.g.*, *Citrus*, *Eucalyptus*, *Quercus*, and *Fagus*). The large sclerotium is formed close to the roots of the host [3], [4] or on basswood that is inoculated and stored in caverns [3]. The large edible sclerotium of *W. cocos* has been referred to as “Indian bread” or “tuckahoe” in North America [3]. *W. cocos* is widely used as nutraceuticals, cosmetics, tea supplements, and functional food [5], [6]. Dried sclerotium, which is known as *Fuling* in traditional Chinese medicine (TCM), is also widely used as a crude drug in East Asia.

Fungal sclerotia are hard, asexual, and long-lived resting structures composed of aggregated vegetative hyphæ [9]. The dry compact biomass of *W. cocos* sclerotium is over 80% of fibers, mainly composed of β-D-glucan-type nonstarch polysaccharides [7], [8]. The developmental mechanisms leading to sclerotial formation have been investigated in *Sclerotinia sclerotiorum*, a model system for the study of sclerotial development and formation [9], [10]. Generally, oxidative stress, low pH, and hyphal damage trigger sclerotial formation [9], [11], [12]. In addition to environmental changes, primary metabolism, secondary messengers, and molecular components also play important roles in regulating sclerotial development [9]. The development of *W. cocos* sclerotium entails complex multistep processes related to peroxisomes, fatty acid desaturation, and degradation pathways [13].

The dried sclerotium of *W. cocos* is a source of many secondary metabolites, including polysaccharides and triterpenoids, which are major ingredients and bioactive compounds of pharmaceuticals [3], [5], [6], [14]. The polysaccharides isolated from polypore fungi with high molecular weight are derived from fungal cell walls [15]. These compounds exhibit anti-inflammatory effects [16], immunomodulatory properties [17], [18], and anticancer activities [5]. A large number of (1-3)-β-D- and (1-6)-β-D-glucan-type polysaccharides and lanostane/secolanostane skeleton-derived triterpenoids have been isolated from *W. cocos* [3], [19]. Although the genome of an American strain of *W. cocos* has been previously released by the Joint Genome Institute (JGI) [20], its features have not been discussed. Regulation of pathways involved in lignocellulose decomposition and triterpenoid synthesis has been investigated [21], [22], [23], but the molecular mechanisms involved in sclerotial development and biosynthesis of secondary metabolites remain largely unknown.

Herein, we report the analysis of the genome and transcriptome of a Chinese strain CGMCC5.78 of *W. cocos*. In addition, we compare this draft genome to the genome of the American strain [20] and to other related fungi (Table S1). Considering the specific feature of the edible and medicinal sclerotia of *W. cocos*, this comparative genome analysis will increase our knowledge about the molecular mechanisms involved in sclerotial formation and bioactive compound biosynthesis in *W. cocos.* Furthermore, these data will open up new avenues for the investigation of genetic breeding and commercial production of the edible and medicinal *W. cocos* sclerotia.

## Results

### Genomic features of *W. cocos*

We sequenced the genome of *W. cocos* (CGMCC5.78) by using HiSeq 2000 Illumina short-reads sequencing and a fosmid-to-fosmid strategy. The size of the final genome assembly is estimated to 50.6 Mb (Table 1, Tables S2 and S3). We mapped the short reads against the genome assembly by SOAPaligner [24] to assess the sequencing depth. The percentage of reads with sequencing depths lower than 10× was less than 2% among the total genomic reads (Figure S1). We also sequenced five randomly selected fosmid clones to assess the accuracy of the genome assembly, and found that their sequences were nearly identical to the corresponding genome scaffolds (Table S4). Additionally, a large number of transposable elements (TEs) were found in the assembled genome, and the most abundant type of TEs was long terminal repeats (LTRs), accounting for ~ 20% of the assembly (Table 2). Moreover, 10,908 gene models were prediected (Table S5), of which 9277 genes (85%) were functionally annotated (Table S6). tRNAs, rRNAs, and snRNAs were also annotated, and 29 of the 184 tRNAs were identified to be pseudogenes (Table S7).

**Table 1:** **Genome characteristics of*W. cocos***

**Genome feature**	**Data**
Coverage	2028
Assembly size (Mb)	50.6
Total contig length (Mb)	48.9
Number of scaffolds (>100 bp)	351
Scaffold L50 (kb)	835
Number of Scaffold N50	16
Number of contigs	1433
Contig L50 (kb)	86.3
Number of Contig N50	171
GC content (%)	51.7
Number of protein-coding genes	10,908
Average gene length (bp)	1829
Average number of exons per gene	5
Average exon size (bp)	268
Average coding sequence size (bp)	1366
Average intron size (bp)	92
Number of tRNA (pseudogenes)	184 (29)
Transposable elements (%)	33.5

**Table 2:** **Repeat content in the assembled *W. cocos*genome**

**Type**	**RepeatMasker**		**ProteinMask**		***De novo***		**Combined**	
	**Length (bp)**	**Genomic content (%)**	**Length (bp)**	**Genomic content (%)**	**Length (bp)**	**Genomic content (%)**	**Length (bp)**	**Genomic content (%)**
DNA element	80,830	0.160	217,077	0.429	914,887	1.807	995,671	1.967
LINE	11,553	0.023	67,062	0.132	509,560	1.007	541,388	1.070
LTR	1,332,164	2.632	2,306,849	4.557	9,965,438	19.688	10,081,749	19.917
SINE	2371	0.005	0	0.000	3886	0.008	4185	0.008
Others	0	0.000	0	0.000	0	0.000	0	0.000
Unknown	214	0.000	0	0.000	6,424,247	12.692	6,424,414	12.692
Total	1,424,019	2.813	2,590,964	5.119	16,788,136	33.166	16,962,149	33.510

*Note*: RepeatMasker and ProteinMask indicate TEs identified by RepeatMasker and ProteinMask pipelines against the database of Repbase (Repbase-16.03), respectively. *De novo* indicates TEs identified by RepeatMasker pipeline against the fasta data of the genome that were generated using PILER-DF, RepeatScout, or LTR_Finder. Combined indicates TEs identified using the three methods mentioned above, with redundant data removed. TE, transposable element; LINE, long interspersed nuclear element; LTR, long terminal repeat; SINE, short interspersed nuclear element.

**Figure 1:**
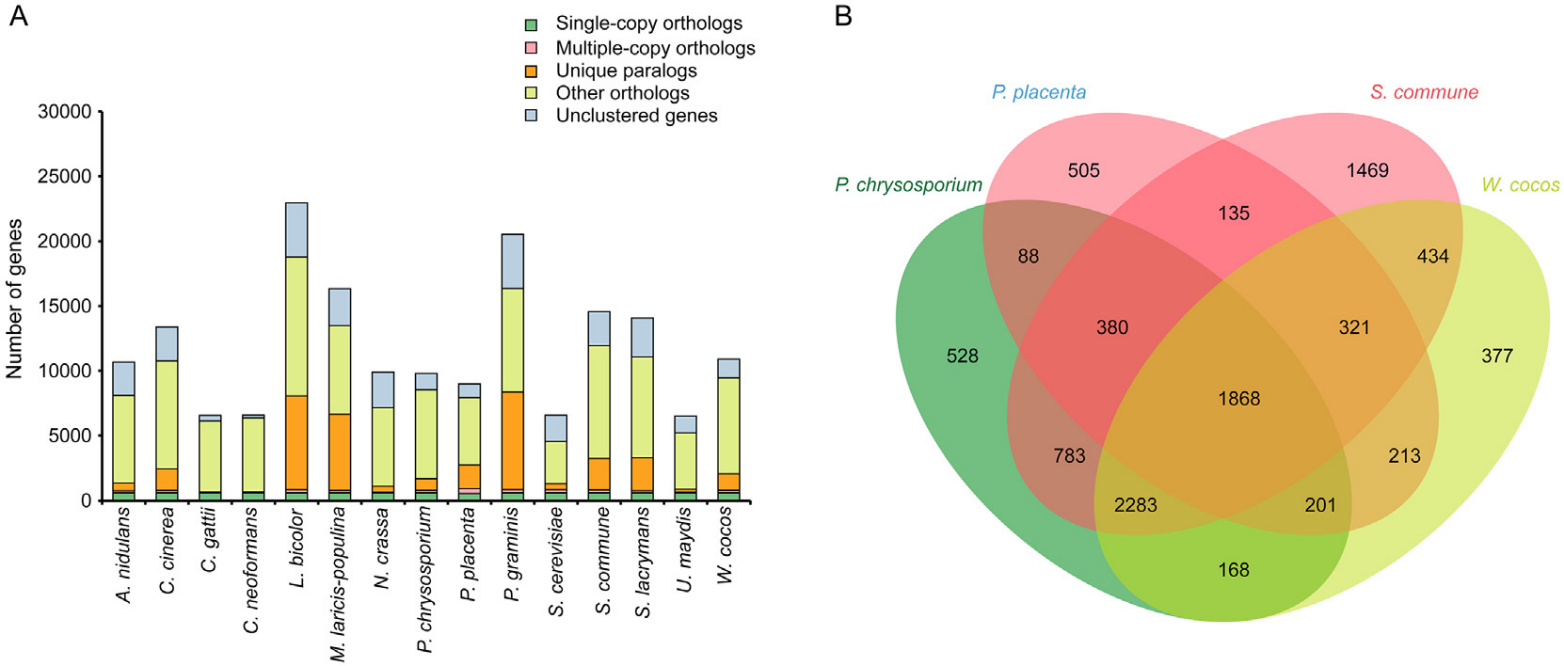
**Genomic features of*W. cocos*** **A.** The number of single-copy orthologs, multiple-copy orthologs, unique paralogs, other orthologs, and unclustered genes in *Aspergillus nidulans*, *Coprinopsis cinerea*, *Cryptococcus gattii*, *Cryptococcus neoformans*, *Laccaria bicolor*, *Melampsora laricis-populina*, *Neurospora crassa*, *Phanerochaete chrysosporium*, *Postia placenta*, *Puccinia graminis*, *Saccharomyces cerevisiae*, *Schizophyllum commune*, *Serpula lacrymans*, *Ustilago maydis*, and *Wolfiporia cocos.***B.** The orthologs identified in *W. cocos*, *P. placenta*, *P. chrysosporium*, and *S. commune* by OrthoMCL after BLASTP (E value < 1E–5)*.*

The numbers and proportions of single-copy orthologs, multiple-copy orthologs, unique paralogs, other orthologs, and unclustered genes in the genomes of *W. Cocos* and other fungi were shown in Figure 1A and Table S8. 1440 species-specific genes and 234 unique gene families were identified in *W. cocos* by comparative genome analysis against other 14 fungi using OrthoMCL [25] (Table S9A). These species-specific genes are enriched in the Gene Ontology (GO) terms of “metabolic process”, “binding”, and “catalytic activity” (Table S9B) and in the Kyoto Encyclopedia of Genes and Genomes (KEGG) pathway of “metabolism” (Table S9C). A total of 1868 orthologs existed in the genomes of *W. cocos*, *Postia placenta*, *Phanerochaete chrysosporium,* and *Schizophyllum commune*, while 377 genes were specific to *W. coco* (Figure 1B).

**Figure 2:**
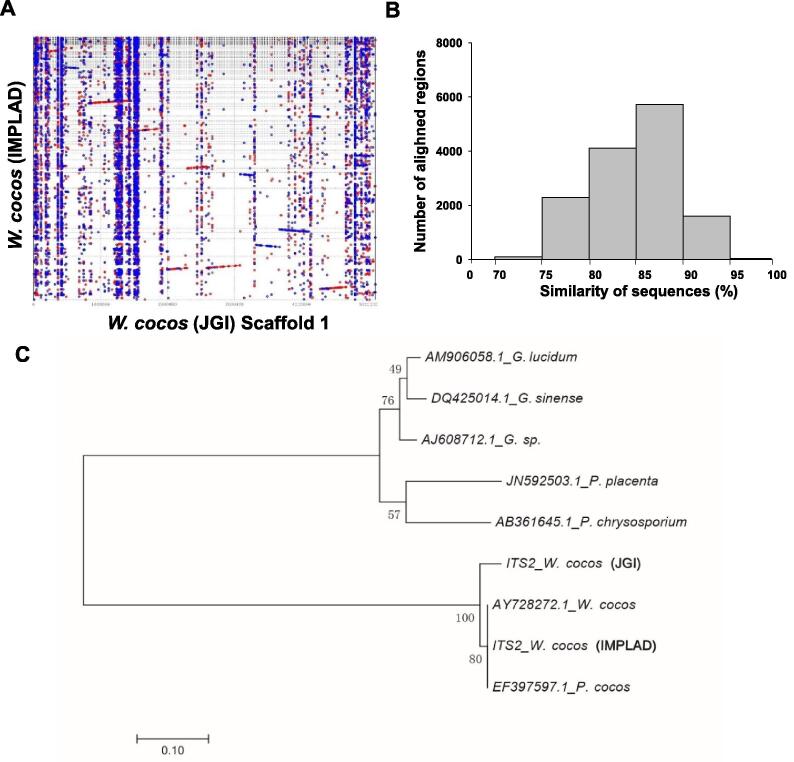
**Genomic comparison of the Chinese and American strains of*W. cocos*** **A.** Synteny dot plot showing the comparison of the whole genome of *W. cocos* (sequenced by IMPLAD in this study) to scaffold 1 of the American *W. cocos* genome (sequenced by JGI). **B.** Histogram showing similarity distribution of the 13,874 aligned regions. **C.** Evolutionary relationships of different *W. cocos* strains with other Polyporales species including *Ganoderma sp*., *Ganoderma lucidum*, *Ganoderma sinense*, *P. chrysosporium*, and *P. placenta* by ITS2 sequences. The sequences of AY728272.1 and EF397597.1 for ITS2 were downloaded from GenBank. The ITS2 sequences of the other two *W. cocos* strains were isolated from the *W. cocos* genomes sequenced by JGI and IMPLAD (CGMCC5.78), respectively. The phylogenetic tree was constructed using the neighbor-joining method. The percentages of replicated trees in which the associated taxa were clustered together in the bootstrap test (1000 replicates) are shown next to the branches. IMPLAD, Institute of Medicinal Plant Development; JGI, Joint Genome Institute.

As expected, the *W. cocos* genomes of the Chinese strain CGMCC5.78 [hereafter referred to as *W. cocos* (IMPLAD)] and the American strain MD-104 SS10 [hereafter referred to as *W. cocos* (JGI)] [20] are highly similar with a high average similarity of 92% for the aligned regions (such as scaffold 1, Figure 2A and B). The number of predicted genes for *W. cocos* (IMPLAD) (10,908 genes) is slightly lower than that of *W. cocos* (JGI) (12,746 genes) [20], possibly due to the use of different gene prediction programs, but also the known heterozygosity of *W. cocos* (IMPLAD)*.* The comparison of OrthoMCL gene families (Table S10) and whole genomes (Table S11) confirmed the genetic differences between these two *W. cocos* strains, as illustrated by the phylogenetic analysis (Figure 2C) and the alignment of the rDNA ITS2 sequences (Figure S2). The number of single-copy orthologs, multiple-copy orthologs, and unique paralogs between these two strains are nearly identical (Figure S3A). A total of 6575 shared orthologous groups were identified in the genomes of these two fungi (Figure S3B). A phylogenetic tree of the genomes of both *W. cocos* strains and the other sequenced fungi (*P. placenta*, *P. chrysosporium*, *Laccaria bicolor*, and *S. commune*) was constructed using single-copy orthologous genes (Figure S3C). The molecular clock for the first phase sites in each species was calculated with single-copy orthologous genes. The divergence time between the two *W. cocos* strains was estimated to be 19.3 million years ago (MYA) (Figure S3C). The difference in gene family expansion and contraction in these strains was also detected by CAFE [26], and the result showed that, of the 6891 gene families presented in the most recent common ancestor of the six fungal species, 154 gene families in *W. cocos* (IMPLAD) exhibited expansion, whereas 1387 genes families exhibited contraction; in comparison, 284 gene families in *W. cocos* (JGI) exhibited expansion, whereas 127 genes families exhibited contraction (Figure S3D). The observed genomic differences might be associated with the phenotypic and physiological differences between the Chinese and American *W. cocos* strains*.*

### Transcript profiling

**Figure 3:**
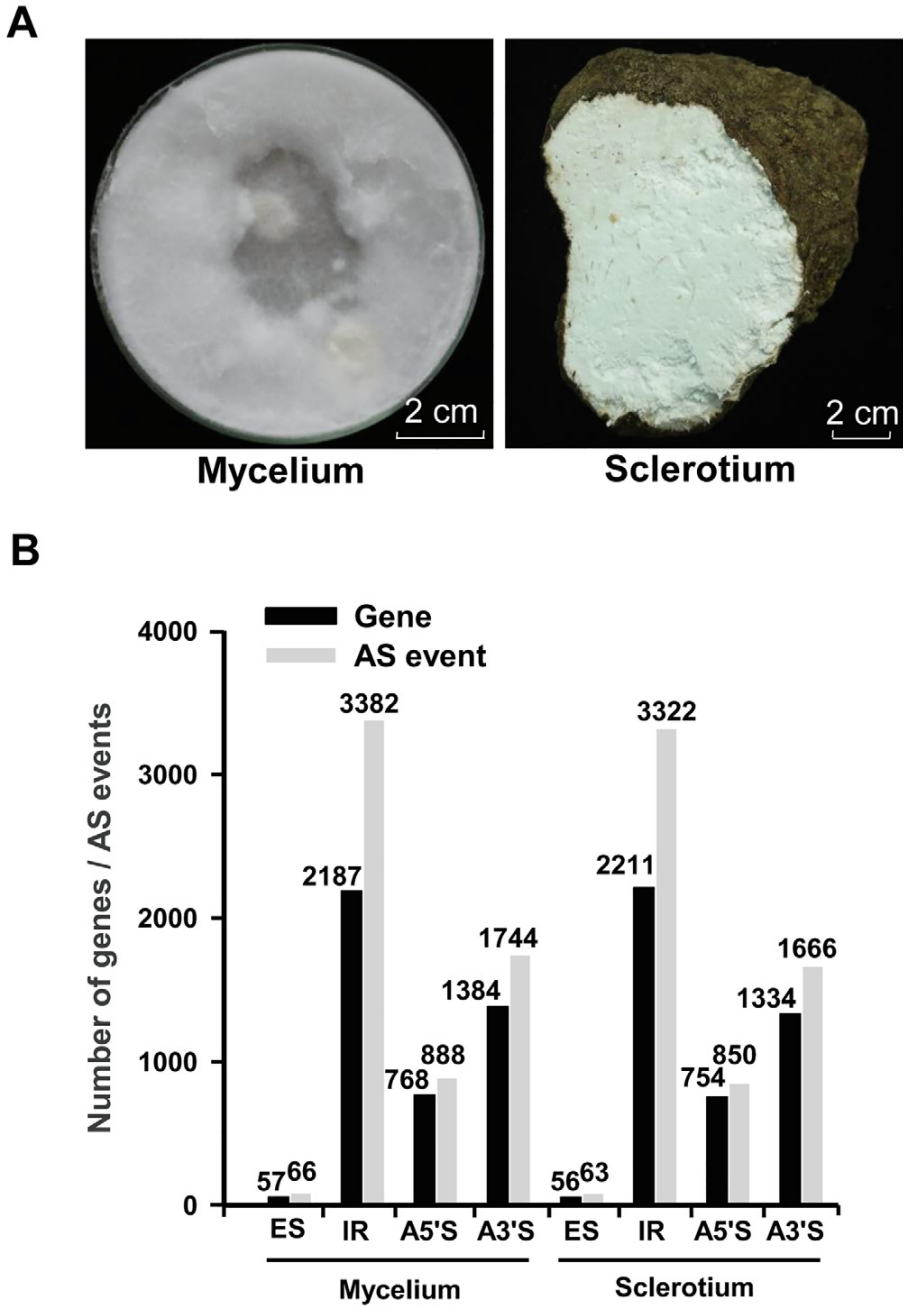
**Transcriptomic analysis of*W. cocos*** **A.** The 7-day-old mycelium and the one-year-old fresh sclerotium of *W. cocos* used for transcriptome analysis. **B.** The number of genes for AS events in the transcriptomes of *W. cocos* mycelium and sclerotium. AS, alternative splicing; ES, exon skipping; IR, intron retention; A5ʹS, alternative 5ʹ splicing; A3ʹS, alternative 3ʹ splicing.

To uncover the molecular mechanisms related to secondary metabolite biosynthesis and sclerotial development, we compared the gene expression profiles between the vegetative mycelium and the sclerotium of *W. cocos* (IMPLAD) (Figure 3A). Among the total RNA reads produced from the mycelium and sclerotium, 73% and 65% of the reads were mapped to the genome, respectively (Table S12). A total of 8548 genes were expressed in the vegetative mycelium (8395 genes) and sclerotium (8479 genes) (Table S13). A relative high sequencing coverage was presented in most of the genes in mycelium and sclerotium (Table S14). A total of 2838 genes showed differential expression in the sclerotium tissues compared to the mycelium tissues: 1877 were upregulated, whereas 961 were downregulated (Table S15). The GO and KEGG enrichment analyses showed that genes involved in “cell/cell part”, “catalytic activity”, “metabolic process” (Table S16), and “metabolic pathways” (Table S17) were prominent. To validate the RNA-seq transcript profiling, the relative expression levels of ten key genes involved in the mevalonate (MVA) pathway were measured by RT-qPCR. The relative expression of seven of these marker genes was consistent with the RNA-seq data (Table S18).

Alternative splicing (AS) events are widespread in the transcriptome of *W. cocos*. Intron retention (IR) and exon skipping (ES) are the largest and least variable AS types in the transcriptomes of vegetative mycelium and sclerotium tissues (Figure 3B, Tables S19 and S20). In addition, a total of 3539 and 3809 novel transcript units (TUs) were predicted in the transcriptomes of the mycelium and sclerotium, respectively (Table S21).

### Genes related to sclerotial development

Gene families of G-protein-coupled receptors (GPCRs), heterotrimeric G protein subunits (G*α* subunit), and monomeric GTPase modules (RhoGEF family), known to be involved in sclerotial development in *S. sclerotiorum* [9], [10], displayed gene expansion in Polyporaceae (*e.g.*, *P. placenta*, *P. chrysosporium*, *Ganoderma lucidum*, and *W. cocos*) compared to Sclerotiniaceae (*e.g.*, *S. sclerotiorum* and *Botrytis cinerea*). In contrast, the histidine kinase gene family is narrowed in Polyporaceae (Table S22). The diversification of these gene families suggests that developmental pathways involved in sclerotial formation in *W. cocos* are likely different to pathogenic sclerotia*.* In the sclerotium of *W. cocos,* the expression of 21 signaling genes was upregulated (*e.g.*, *Gα* subunit, *RasGEF*, *RhoGEF*, *RhoGAP*, *MAPK*, and *MAPKK*), whereas the expression of 13 was downregulated (*e.g.*, *STE3*, *Gα* subunit, *RGS*, and *RhoGEF*) compared to that in the mycelium, suggesting that they play a key role in the developmental transition from mycelium to sclerotium*.* In contrast, orthologous genes involved in metabolic pathways were shared between *W. cocos* and sclerotial-producing fungi *S. sclerotiorum* and *B. cinerea* (Table S23), indicating that several metabolic processes have been co-opted in sclerotia produced by taxonomically divergent species.

### *Genes related to the mating process*

The mating process in Basidiomycota is coordinated by key homeodomain (*HD*) genes and pheromone recepoter genes [27]. A candidate *HD1-HD2* gene pair (WCO006754.1-WCO006755.1) was annotated in *W. cocos* (IMPLAD) genome (Figure S4 ; Table S24). These two *HD* genes were located in scaffold 4, 73 kb away from a conserved mitochondrial intermediate peptidase gene (*MIP*; WCO006733.1) (Figure S4; Table S24), while a conserved fungal gene of unknown function (*β-FG*, WCO000491.1) was located in scaffold 1 (Table S24). Interestingly, another *HD*-like gene *HD3* (WCO006732.1) was located near the *MIP* gene in scaffold 4. In contrast, a *MIP* gene and a *β-FG* gene were both located in scaffold 1 and no *HD* genes were identified in *W. cocos* (JGI) genome (Figure S4; Table S24). Furthermore, two STE3-like pheromone receptor genes (WCO006868.1 and WCO007080.1) existed in different scaffolds of *W. cocos* (IMPLAD) genome. Similarly, four STE3-like pheromone receptor genes were also located in different scaffolds of *W. cocos* (JGI) genome (Figure S4; Table S24).

#### Genes related to fruiting body formation

A white or slightly yellow structure, similar to a honeycomb, is the sexual reproductive organ of *W. cocos* (Figure S5). This fruiting body usually grows on the surface of wood, soil, or culture media and produces spores. The morphological characteristics of the fruiting body are the basis for the classification of *W. cocos*. According to a previous study on the formation of fruiting bodies in other fungi [10], the signalosome including sexual development subunits, transcription factors (TFs), mutanase, fatty acid oxygenase, and transcriptional activators might play a role in the development and formation of fruiting bodies. The homologous genes of these components have been identified in *W. cocos* genome (Table S25).

### Genes involved in secondary metabolism

#### Genes related to polysaccharide biosynthesis and regulation

**Figure 4:**
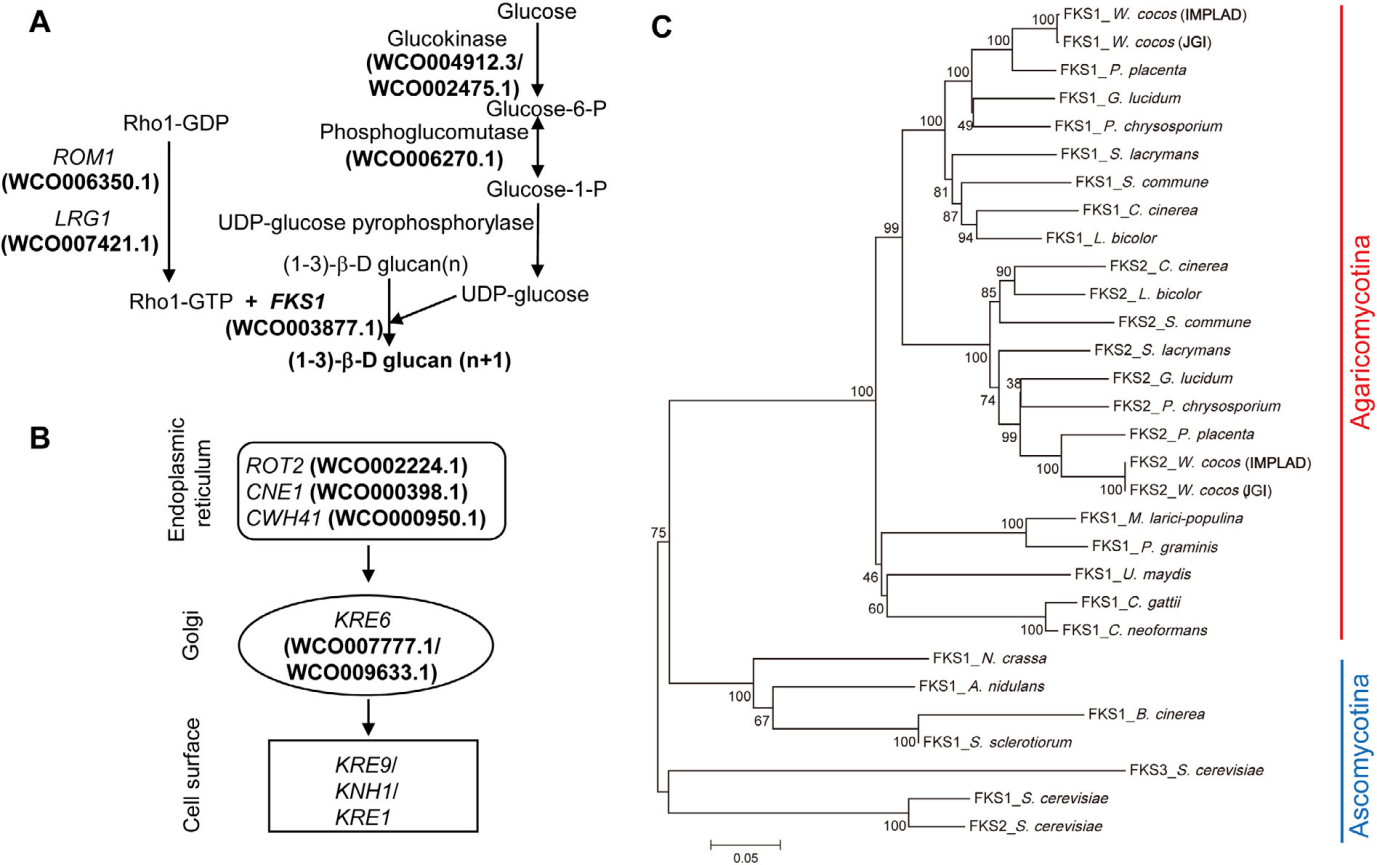
**The putative biosynthetic genes and regulatory components of (1-3)-β-D and (1-6)-β-D glucan (polysaccharide)biosynthesis** **A.** The predicted biosynthetic genes involved in (1-3)-β-D glucan synthesis in *W. cocos*. **B.** The putative biosynthetic steps involved in (1-6)-β-D glucan synthesis in *W. cocos*. **C.** The phylogeny of glucan synthase (FKS) proteins in *W. cocos* and other fungi, including of *W. cocos* (IMPLAD), *W. cocos* (JGI), *P. placenta*, *P. chrysosporium*, *G. lucidum*, *S. lacrymans*, *S. commune*, *L. bicolor*, *C. cinerea*, *P. graminis*, *M. larici-populina*, *U. maydis*, *C. neoformans*, *C. gattii*, *N. crassa*, *A. nidulans*, *Sclerotinia sclerotiorum*, *Botrytis cinerea*, and *Saccharomyces cerevisiae.* The two FKSs (FKS1 and FKS2) identified from *W. cocos* sequenced by IMPLAD and JGI, respectively, showed the highest homology among all the FKSs. *ROM1*, *RHO1* multicopy suppressor; *RHO1*, Ras homologous 1; *LRG1*, LIM-RhoGAP homologous gene 1; *FKS1*, FK506-supersensitive 1; *ROT2*, reversal of TOR2; *CNE1*, calnexin; *CWH41*, calcofluor white hypersensitive 41; *KRE6*, killer toxin resistant 6; *KRE9*, killer toxin resistant 9; *KNH1*, KRE9 homolog; *KRE1*, killer toxin resistant 1.

In *W. Cocos*, (1-3)-β-D and (1-6)-β-D glucans are the major components of the bioactive polysaccharides, and their concentrations impact the quality and value of *W. cocos* sclerotia. The biosynthesis of these glucans was extensively studied in yeast [28], [29]. (1-3)-β glucans are synthesized from UDP-glucose by the (1-3)-β glucan synthase (GS) complex (EC:2.4.1.34) [30], which is composed of a catalytic subunit (Fksp, encoded by *FKS1* [31], [32]) and a regulatory subunit (Rho1p) [33] in *Saccharomyces cerevisiae*. Genes involved in biosynthesis of (1-3)-β-D and (1-6)-β-D glucans are depicted in Figure 4A and B, together with a phosphoglucomutase gene (WCO006270.1) that is highly expressed in the sclerotium (Table S26).

Fksp (EC: 2. 4.1.34) uses UDP-glucose to produce a linear polysaccharide [28]. There are two glucan synthase genes, designated as *FKS1* (WCO003877.1) and *FKS2* (WCO000998.1), in the *W. cocos* (IMPLAD) genome; however, only *FKS1* displayed differential expression during sclerotial development (Table S26). *FKS1* was upregulated in sclerotium compared to mycelium, indicating its essential role in the regulation of polysaccharide biosynthesis and sclerotial development in *W. cocos* (Table S26). Phylogenetic analysis of FKS proteins suggests that this enzyme is highly conserved in fungi and, as expected, FKS from the Chinese and American strains showed the highest homology (Figure 4C; Table S27).

The genes responsible for the regulation of *S. cerevisiae* cell wall polysaccharide content have been characterized [28], [29]. Several of these genes and gene families were likely involved in the regulation of polysaccharide biosynthesis in *W. cocos,* including *RHO1* multicopy suppressor (*ROM1*; WCO006350.1) and killer toxin resistant 6 (*KRE6*; WCO007777.1 and WCO009633.1) (Table S26)*.* Moreover, these three genes were highly expressed in the sclerotium, which might be relevant to the higher polysaccharide content in sclerotium.

#### Genes related to triterpenoid biosynthesis

**Figure 5:**
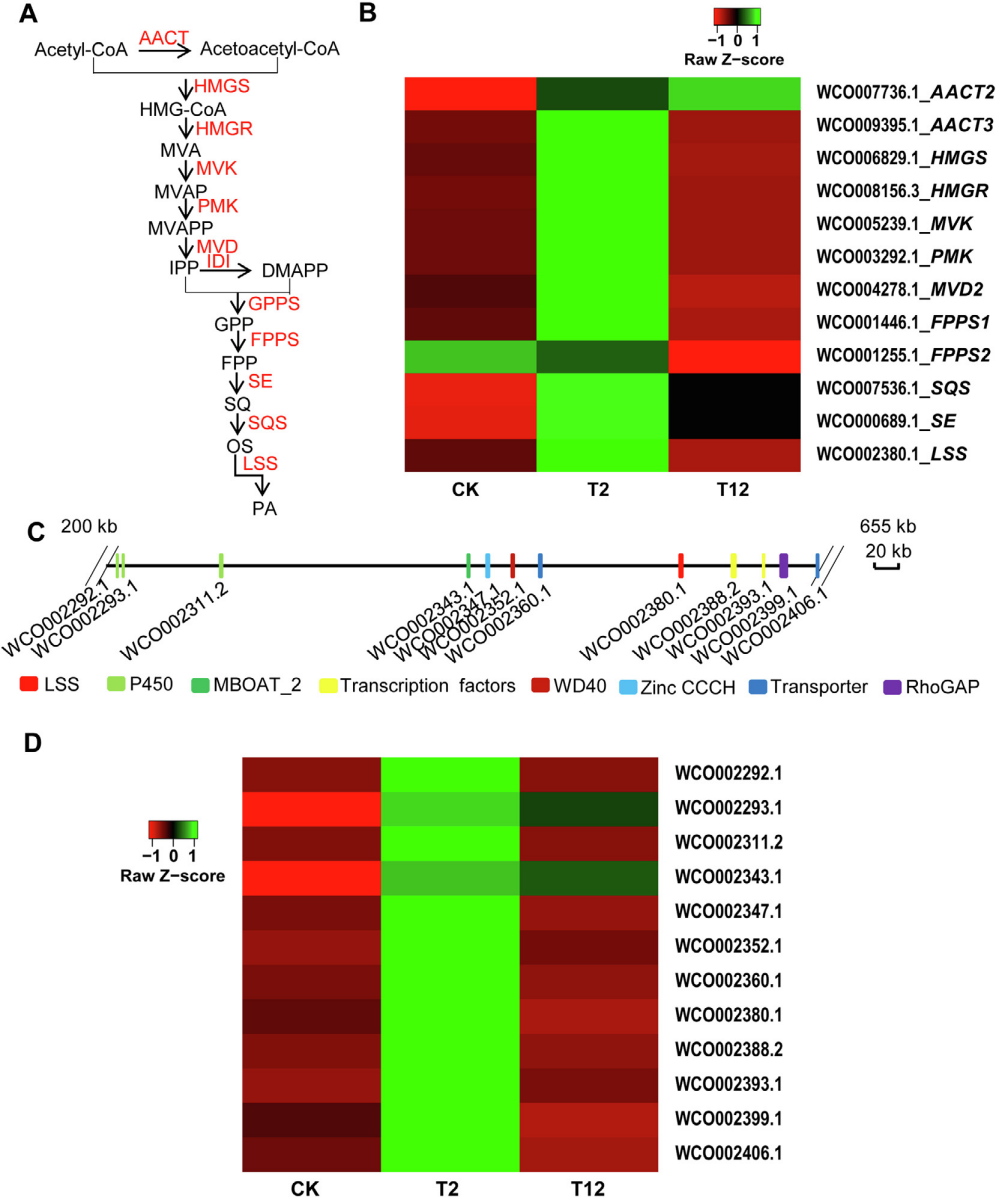
**Biosynthesis of triterpenoids inW. cocos** **A.** The putative biosynthetic pathway of PA, a lanostane-type triterpenoid in *W. cocos* with diverse bioactivities that is biosynthesized via the MVA pathway. All the genes encoding the predicted enzymes invovled in the MVA pathway have been identified in *W. cocos* (IMPLAD) genome. **B.** The expression patterns of the genes encoding enzymes involved in the MVA pathway induced by MeJA (200 μM) treatment for 2 h (T2) and 12 h (T12) compared to the control group (CK). **C.** The potential gene cluster including 12 genes for triterpenoid biosynthesis identified in *W. cocos* (IMPLAD) genome. These genes encoded P450s, transcription factors, transporters, and LSS. **D.** Coexpression analysis of the genes belonging to the *LSS* gene cluster induced by MeJA (200 μM) treatment for 2 h (T2) and 12 h (T12) compared to the control group (CK). AACT, acetyl-CoA acetyltransferase; HMGS, hydroxymethylglutaryl-CoA synthase A; HMGR, 3-hydroxy-3-methylglutaryl-coenzyme A reductase; MVK, mevalonate kinase; PMK, 5-phosphomevalomate kinase; MVD, diphosphomevalonate decarboxylase; IDI, isopentenyl diphosphate isomerase; GPPS, geranyl diphosphate synthase; FPPS, farnesyl diphosphate synthase; SE, squalene monooxygenase; SQS, squalene synthase; LSS, lanosterol synthase; PA, pachymic acid.

Pachymic acid (PA), a lanostane-type triterpenoid with a wide range of bioactivities, is synthesized via the mevalonate (MVA) pathway (Figure 5A). All genes involved in the MVA pathway were annoated in *W. cocos* genome, including several gene families [*e.g.*, acetyl-CoA acetyltransferase (*AACT*) family, farnesyl diphosphate synthase (*FPPS*) family, and diphosphomevalonate decarboxylase (*MVD*) family], and single-copy genes [*e.g.*, 3-hydroxy-3-methylglutaryl-coenzyme A reductase (*HMGR*), hydroxymethylglutaryl-CoA synthase A (*HMGS*), lanosterol synthase (*LSS*), and squalene monooxygenase (*SE*)] (Table S28). Upregulation of most of these genes was induced by methyl jasmonate (MeJA) for 2 h treatment and then downregulation occurred after 12 h treatment (Figure 5B); however, the expression of *AACT1* (WCO010060.1), *FPPS3* (WCO002210.1), *MVD1* (WCO001214.1), *MVD3* (WCO006131.1), and *MVD4* (WCO006124.1) were not detected in this assay (data not shown). In addition, transcriptome analysis showed that *AACT1* (WCO010060.1), *HMGR* (WCO008156.3), *HMGS* (WCO006829.1), *MVD2* (WCO004278.1), and *SE* (WCO000689.1) were more highly expressed in mycelium than in sclerotium, implying the important function of these genes in triterpenoid biosynthesis in *W. cocos* (Table S28).

Lanosterol is the first cyclic intermediate for PA biosynthesis and is catalyzed by the rate-limiting enzyme LSS. A potential gene cluster involved in triterpenoid biosynthesis was located in scaffold 16 (Figure 5C; Table S29). The expression of 22 genes belonging to this cluster was measured in the mycelium incubated in the presence of MeJA (Table S29). Two *CYP450* genes (WCO002292.1 and WCO002311.2), two TF genes (WCO002388.2 and WCO002393.1), one CCCH Zinc finger gene (WCO002347.1), one WD40 domain containing gene (WCO002352.1), and two transporter genes (WCO002360.1 and WCO002406.1) were significantly co-expressed with *LSS* (WCO002380.1) (Figure 5D), providing support for the predicted function of this gene cluster in triterpenoid biosynthesis in *W. cocos*.

#### Genes related to other secondary metabolites

Genome mining revealed multifunctional modular polyketide synthases (PKSs) and nonribosomal peptide synthetases (NRPSs) that are responsible for the biosynthesis of most biologically-active metabolites in microorganisms [34]. Although previous studies have not identified any products of PKSs or NRPSs in *W. cocos* [3], [35], [36], a total of eight genes encoding NRPS-like proteins, seven genes encoding PKS proteins, and eight genes encoding PKS/NRPS-like proteins were identified in the present study (Table S30A). Similarly, 13 predicted gene clusters containing these core genes were predicted by antiSMASH annotation [37] (Table S30B and C), including five *NRPS-like* gene clusters and eight *PKS* gene clusters. These gene clusters consisted of 3–18 genes, implying that some clusters are incomplete. In addition, 15 terpene gene clusters were also discovered (Table S30B and C). These gene clusters might participate in the production of terpenes.

#### Genes related to cell wall degradation

To identify enzymes involved in cell wall degradation, *W. cocos* genes were searched against the carbohydrate-active enzymes (CAZymes) database [38]. A total of 321 genes encoding CAZymes were predicted in the *W. cocos* (IMPLAD) genome, including 139 glycoside hydrolase (*GH*) genes, 63 glycosyl transferase (*GT*) genes, 58 carbohydrate esterase (*CE*) genes, 20 carbohydrate-binding module (*CBM*) genes, 38 auxiliary activity (*AA*) genes, and 3 polysaccharide lyase (*PL*) genes (Table S31A). Most CEs, GHs, and AAs are involved in the hydrolysis of carbohydrate and noncarbohydrate substrates and the oxidative degradation of lignin-based components of the plant cell wall.

The presence of multiple gene copies for *GH5*, *GH16*, *GH18*, and *GH28*, which were involved in the degradation of cellulose, β-glucan, chitin, and pectin, respectively, and the absence of *GH11* (encoding a xylanase) and *CE12* (encoding an acetylesterase) in *W. cocos* (IMPLAD) are consistent with a previous study [39]. The family 1 carbohydrate binding modules (*CBM1*), which is generally absent in brown-rot fungus, was also absent in *W. cocos* (IMPLAD). Compared with *P. placenta* and *Fomitopsis pinicola*, the gene numbers of *GHs* in both *W. cocos* (IMPLAD) and *W. cocos* (JGI) were similar to those in *P. placenta* but less than that of *F. pinicola* (Table S31B and C). CEs hydrolyze a wide range of carbohydrate and noncarbohydrate substrates. The *W. cocos* (IMPLAD) genome covered a higher number of *CE* family (58 genes) compared to other brown-rot species of fungi in this study. In addition, 83 *CAZyme* genes were upregulated and 48 genes were downregulated in sclerotium compared to those in mycelium (Table S31A). The most abundantly upregulated genes in sclerotium were in the *CE10* family (7 genes), followed by the *GH16* (5 genes), *GH13* (5 genes), and *GH5* (4 genes) families.

#### Genes encoding membrane transporters

A total of 3710 transporters, belonging to 85 families, was predicted in *W. cocos* (IMPLAD), accounting for 34% of the total predicted genes (Table S32). The ATP-binding cassette (ABC) transporters were the most abundant category, together with 94 major facilitator superfamily (MFS) transporters, which may play key roles in the transportation of secondary metabolites in *W. cocos*. A total of 162 upregulated and 165 downregulated genes were detected in sclerotium compared to mycelium, indicating more active transportation in cells during sclerotial development and formation. It is worth noting that most of the significantly upregulated genes in sclerotium were members of the ABC gene superfamily, whereas most of the downregulated genes encode glycoside-pentoside-hexuronide (GPH) proteins, a cation symporter family, suggesting the different functions of these transporters in *W. cocos*.

### Regulation of secondary metabolism and fungal development

#### Velvet protein family

The velvet protein complex plays essential roles in the regulation of fungal development and secondary metabolite production in Fungi [40], [41]. Ten genes encoding velvet domain-containing proteins were identified in *W. cocos* (Figure S6; Table S33). In particular, *VosA* (WCO008289.1) and *VelB* (WCO005599.1) showed significantly upregulated expression patterns in sclerotium. Several *velvet* genes were distributed in clusters with a head-to-tail structure, such as WCO009956.3–WCO009957.1, WCO000954.1–WCO000955.1, and WCO010849.1–WCO010850.1. All these velvet proteins have homologs in *W. cocos* (JGI) (Table S33).

#### Transcription factors

In total, 307 genes encoding TFs were predicted in *W. cocos* (IMPLAD) genome, with the most abundant family being the zinc knuckle TF family (130 genes), followed by the fungal-specific TF-domain family (43 genes) and the fungal Zn_(2)_-Cys_(6)_ binuclear cluster domain family (29 genes) (Table S34). 69 TFs were differentially expressed between the transcriptomes of sclerotium and mycelium, including prominent TF genes in the fungal-specific TF-domain family and the fungal Zn_(2)_-Cys_(6)_ binuclear cluster domain family, suggesting the suspected roles of these TFs in the regulation of fungal development in *W. cocos*.

## Discussion

Herein, we characterized the genome and transcriptome of the Chinese strain CGMCC5.78 of *W. cocos* to investigate the molecular mechanisms related to the formation of the edible and medicinal sclerotium. We identified several differentially expressed genes (DEGs) that may be involved in sclerotial development and characterized metabolic pathways that are involved in the biosynthesis of several bioactive secondary metabolites, such as triterpenoids and glucans.

The present comparative genome analysis also uncovered substantial genetic variations between the Chinese (CGMCC5.78) and American (MD-104 SS10) strains of *W. cocos* (Figure 2, Figure S2), which are consistent with the polymorphism observed between another Chinese and a Japanese *W. cocos* strains [42]. Transcript profiling of the vegetative mycelium and sclerotium (Table S15) showed that a striking metabolic reorganization is induced by the mycelium-to-sclerotia transition. The AS events (Figure 3B; Tables S19 and S20) and novel TUs (Table S21) provide useful information for further research on gene characterization in *W. cocos*.

The molecular basis of sclerotial formation has been elaborated in *S. sclerotiorum* [9], [10]. *W. cocos* possesses a similar genomic capacity for the formation of its edible and medicinal sclerotium. Sclerotial development comprises multiple processes and is affected by environmental changes (*e.g.*, oxidative stress and low pH), primary metabolism (*e.g.*, fatty acid desaturation and degradation pathways), secondary messengers, and signaling components [43], [44], [9], [10], [11], [12]. Large sclerotia of *W. cocos* can be induced by an early inoculation of small sclerotia on the medium [45], indicative of a specific signal transmitted from the small sclerotia for the induction of larger sclerotia. Signaling factors (*e.g.*, pH-responsive TFs, Pac1, heterotrimeric G proteins, MAP kinases, Ras, protein phosphatases, and adenylate cyclases) might be responsible for the revulsive cultivation of sclerotia (Table S22). The principal difference in the genomic repertoire involved the expansion of the heterotrimeric G protein subunits (*e.g.*, G*α* subunit) and the monomeric GTPase modules (*e.g.*, RhoGEF proteins), which represent the candidate sclerotium-associated factors in *W. cocos*.

Sclerotial formation begins with the degradation of plant cell walls and relies on the colonization of woody tissues [46]. A sustainable carbon source is one of the nutritional requirements for sclerotial development [39]. *W. cocos* decomposes celluloses and hemicelluloses of *Pinus* species early in the decay process, similar to other brown wood-decaying fungi, to provide the carbon required for substantial sclerotial development and formation [46]. CAZymes (Table S31) contribute to the degradation of pine wood tissues and release carbohydrates and other unknown nutrients that are absorbed and utilized by *W. cocos* to form sclerotia [39]. *W. cocos* genome is enriched in *CE* genes (58 genes) (Table S31). This CAZyme repertoire differs from other brown-rot fungi and may be related to the specific habitat of *W. cocos*.

Previous studies showed that the mating type of *W. cocos* appears to be bipolar [47], [48], and the primary and secondary hyphae are without clamp connections [46], [48]. In the mating process, *HD* genes and pheromone receptor genes are physically linked and localized in one mating-type locus (*MAT-A* locus) in bipolar species. In tetrapolar species, these two types of genes are located in two unlinked loci and designated as the *MAT-A* (or *HD*) locus and *MAT-B* (or pheromone receptor gene) locus [49], [50]. The *MAT-A* locus genes are organized with a highly conserved gene order that includes one or more pairs of *HD1* and *HD2* [51], with an *MIP* and a *β-FG* flanking the *HD1-HD2* gene pair(s) [47]. The *HD1-HD2* gene pair of *W. cocos* was located in the same scaffold with an *MIP*, while the *β-FG* was found in another scaffold (Figure S4A; Table S24). This may result from the assembly fragmentation. The *HD* and pheromone receptor genes are present in all Agaricomycete genomes, regardless of whether they are bipolar or tetrapolar [49]. Consequently, two STE3-like pheromone receptor genes (WCO006868.1 and WCO007080.1) belonging to the *MAT-B* locus were also identified in the *W. cocos* genome (Table S24). These mating locus genes provide molecular evidence for the determination of the mating process of *W. cocos*.

The biosynthesis of secondary metabolites is often coupled with morphological development in fungi [52]. Several polysaccharides and triterpenoids are bioactive compounds that are responsible for the medicinal function of *W. cocos.* Bioactive polysaccharides comprise dietary fibers accumulating in sclerotium, including a structurally diverse class of biological macromolecules in the cell wall with wide-ranging physicochemical properties [3]. Linear (1-3)-β-D glucan is the primary constituent of the polysaccharides isolated from the *W. cocos* sclerotium [53]. Because molecular components involved in the biosynthesis and regulation of polysaccharides in cell wall have been characterized in yeast [28], [29], we were able to identify the homologous genes in *W. cocos* (Figure 4; Table S26), providing the most likely candidate genes invovled in polysaccharide biosynthesis and regulation in *W. cocos.*

Although several MVA pathway-related genes have been identified in *W. cocos* [21], the present *W. cocos* genome provides the entire collection of genes and a potential gene cluster participating in PA and triterpenoid biosynthesis (Figure 5; Table S28). The gene cluster is similar to a typical terpene biosynthetic gene cluster; it contains the *LSS* signature gene that is clustered with genes coding for TFs and transporters that likely correspond to tailoring enzymes and components (Figure 5C; Table S29) [54], [55]. Moreover, most of the genes in this cluster were coexpressed with *LSS* after MeJA induction, which conforms to the characteristics of a gene cluster (Figure 5D). Intriguingly, this gene cluster has not been identified in the *G. lucidum* genome [56], inferring the specific function of the gene cluster for PA biosynthesis in *W. cocos.*

The expression of biosynthetic genes related to secondary metabolites is governed by a network of key regulators that respond to diverse environmental cues. The velvet proteins, a class of key regulators, can coordinate the crosstalk of secondary metabolism and differentiation processes, such as asexual or sexual sporulation and sclerotium or fruiting body formation [41]. In *W. cocos,* the *velvet* gene family (ten members) is expanded compared to *B. cinerea*, *S. sclerotiorum*, *P. chrysosporium*, and *G. lucidum* (Table S33). This gene expansion might be due to tandem duplication of *velvet* genes in *W. cocos*. In addition, the signaling components of G proteins and other regulators (including TFs) (Table S34) also regulate the crosstalk of secondary metabolism and fungal development [52], [57]. This molecular information will be helpful in the illustration of the regulatory mechanisms controlled by signaling components in *W. cocos.*

## Conclusion

The present *W. cocos* genome and transcriptome resources will provide novel opportunities for investigating the mechanisms driving the formation of edible and medicinal sclerotia in fungi. This study also provides new insights into the diversification of the molecular mechanisms involved in fungal development and secondary metabolism in *W. cocos*. We identified several DEGs related to signaling components, secondary metabolism, nutrient transport, TFs, mating process, and CAZymes that are likely involved in the sclerotial formation (Figure S7). Finally, we have identified several genes involved in the biosynthesis of key secondary metabolites that are relevant to the medicinal properties of the fungus.

## Materials and methods

### Fungal strain and culture conditions

*W. cocos* dikaryotic strain (CGMCC5.78) was obtained from the China General Microbiological Culture Collection Center (Beijing, China, http://www.cgmcc.net/) and stored in our laboratory. This is one of the most widely used strains in China. Vegetative mycelia were grown on potato dextrose medium in the dark at 28 °C. Liquid cultures for the mycelia were shaken at 50 rpm for 7 days. The mature sclerotia of the strain CGMCC5.78 used for transcriptome analysis were cultivated on *Quercus variabilis* Blume logs at HuiTao Pharmaceutical Company (Luotian, China).

### Construction of the fosmid library

High-molecular-weight (HMW) DNA was isolated from 7-day cultured mycelia using the CTAB DNA isolation method. Genomic DNA was randomly sheared to approximately 40 kb in size. Then, the sheared molecules were size-selected using Pulse Field Gel Electrophoresis (Bio-Rad, Hercules, CA). The sheared DNA was ligated into the pCC2FOS vector (Epicentre, Madison, WI) after end-repair. The ligations were packaged using MaxPlax Lambda Packaging Extracts (Epicentre). The lambda phages carrying foreign DNA were used to infect EPI300-T1R *E. coli* cells (Epicentre). Positive fosmid clones were selected using lysogeny broth (LB)-chloramphenicol (12.5 μg/ml) plates. A total of 11,232 clones were selected and transferred to 96-well plates containing frozen LB media and stored at −80 °C.

### ***De novo*** genome sequencing and assembly

A strategy combining fosmid construction, fosmid shotgun sequencing, and assembly was used in this study. In total, we obtained 11,232 fosmid clones from the fosmid library. Different indices and adapters were added to the fosmid clones to construct the sequencing library. The clones were pooled and sequenced by using HiSeq 2000 (Illumina, San Diego, CA). Finally, we obtained 178.44 Gb data. In addition, we constructed genomic libraries with 511 bp, 2 kb, 5 kb, 10 kb, and 20 kb in length and used the paired-end information to construct contigs and longer scaffolds. A set of exclusion steps was included following preprocessing. These steps included the removal of reads with low-quality bases > 40 bp or reads with *N* base content of more than 10% or poly A. The overlapping reads (read1 and read2 overlap at least 10 bp and mismatch less than 10%) and duplicate reads (the sequence of read1 was equal to read2, which means duplication) were also excluded. After removal of the vector sequence from fosmid clones, the reads generated from the HiSeq 2000 were used in assembly. The short reads were first assembled into fosmid clone sequences using SOAP *de novo* [24]. After removing the remaining vector sequences and other low-quality bases, the fosmid clone sequences were assembled into contig sequences using Celera software. The redundant sequences were first removed via BLAST by alignment to the National Center for Biotechnology Information (NCBI) microbial nucleotide database for any alignment query contigs. Then, the redundant sequences based on the *K*-mer frequencies obtained from the short library reads were removed. Finally, the contig sequences were connected into scaffold sequences by using SSPACE (version 1.1) software. The assembled contig sequences were manually corrected to build the finished scaffolds.

### Gene prediction and annotation

*De novo* gene prediction was executed using Augustus, GeneMark-ES, and SNAP based on the Hidden Markov Model (HMM). Additionally, the homolog-based method was used to weight and calibrate gene structure. The combination of *de novo* gene prediction and gene structure prediction was then used to obtain the GLEAN gene groups. The GLEAN data were corrected by RNA-seq data to accomplish gene prediction. Genes were functionally predicted by BLAST against the public databases, including NCBI nonredundant protein database (Nr), NCBI nonredundant nucleotide database (Nt), InterProScan, GO database, KEGG database, Clusters of Orthologous Groups (COG) database, and TrEMBL/Swissprot database. The PFAM families of TFs in fungi were selected to search the *W. cocos* genome [58]. The Transporter Classification Database (TCDB; http://www.tcdb.org) [59] were used to designate transporters in the *W. cocos* genome by BLAST (E-value = 1E–10).

### Identification of TEs and noncoding RNA

TEs were searched in the *W. cocos* genome using RepeatMasker and ProteinMask software based on Repbase (16.03) data. The *de novo* genome repeat sequence database was then constructed by PILER-DF, RepeatScout, and LTR-Finder, and redundant sequences were removed. TEs were identified with the RepeatMasker software in the clean *de novo* genome repeat sequence database. tRNAs were identified by tRNAscan-SE software. rRNAs were identified by BLAST against the rRNA database of *S. commune*.

### Ortholog analysis

Orthologous groups from the related fungal genomes of *Coprinopsis cinerea*, *Cryptococcus gattii*, *Cryptococcus neoformans*, *Aspergillus nidulans*, *L. bicolor*, *Melampsora laricis-populina*, *Neurospora crassa*, *P. chrysosporium*, *Puccinia graminis*, *S. cerevisiae*, *S. commune*, *Serpula lacrymans*, and *Ustilago maydis* were identified using OrthoMCL [25] version 2.0 (http://www.orthomcl.org). The cutoff of BLASTP was set as E-value < 1E–5.

### Transcriptome sequencing and analysis

Transcriptome analyses were performed on the materials obtained from the 7-day-old mycelia and mature sclerotia of *W. cocos*. Frozen samples were ground to powder in liquid nitrogen for RNA isolation. Total RNA from each sample was extracted using an RNeasy Plant Mini Kit (Qiagen, Dusseldorf, Germany) according to the manufacturer’s instructions. These total RNA samples were also reverse-transcribed into cDNA to validate the RNA-seq data by RT-qPCR method. Genomic DNA was eliminated from the total RNA using DNase I (NEB, Ipswich, MA). RNA integrity and quality were tested using the RNA 6000 Nano II kit on a Bioanalyzer 2100 (Agilent Technologies, Lexington, MA). The RNA samples were sequenced using an Illumina HiSeq 2000 (Illumina). RNA-seq data from the mycelial and mature sclerotial tissues were assembled into transcripts using Trinity. Transcript abundance was estimated by the RPKM method [60]. Differential expression analysis was executed according to the method reported by Audic and Claverie [61]. The AS events were analyzed by TopHat software [62].

### RT-qPCR analysis of gene expression

Total RNA was extracted from 10-day-old *W. cocos* mycelia under the treatment of 200 μM MeJA for 0 h (CK), 2 h (T2), and 12 h (T12), respectively. The RNA was then treated with recombinant DNase I (Life Technologies, Burlington, Canada) at a concentration of 1.5 U/μg total RNA. Single-strand cDNA was synthesized using the PrimeScript™ 1^st^ Strand cDNA Synthesis kit (TaKaRa, Shlga, Japan), 1 μg RNase-free DNase I-treated (TaKaRa) total RNA, and random primers. RT-qPCR was performed at least three times for each sequence using SYBR®Premix Ex TaqTM (Perfect Real Time) (TaKaRa) and an ABI PRISM 7500 real-time PCR System (Life Technologies). Each reaction contained 7.5 μl 2× SYBR Green Master Mix Reagent (Life Technologies), 1.0 μl (10 ng) cDNA, and 200 nM gene-specific primers in a total reaction volume of 15 μl. The PCR amplification program consisted of denaturation at 95 °C for 30 s followed by 40 cycles of 95 °C for 5 s and 60 °C for 34 s. The relative gene expression data were normalized against an internal reference gene, cyclophilin (*CYP*) [23]. The relative expression levels were calculated by the 2^−ΔΔCt^ method. The primers were designed using Primer3 (http://frodo.wi.mit.edu/primer3/). The primers used for RT-qPCR in this study are listed in Table S35.

## Data availability

The raw data for the  genome and transcriptome of the Chinese strain CGMCC5.78 *W. cocos* have been deposited in the Genome Sequence Archive [63] at the National Genomics Data Center, Beijing Institute of Genomics, Chinese Academy of Sciences / China National Center for Bioinformation (GSA: CRA003688), and are publicly accessible at http://bigd.big.ac.cn/gsa.

## CRediT author statement

**Hongmei Luo:** Writing - original draft, Writing - review & editing, Formal analysis. **Jun Qian:** Software, Visualization. **Zhichao Xu:** Formal analysis. **Wanjing Liu:** Validation, Data curation. **Lei Xu:** Resources, Investigation. **Ying Li:** Formal analysis. **Jiang Xu:** Investigation, Data curation. **Jianhong Zhang:** Validation, Data curation. **Xiaolan Xu:** Validation, Data curation. **Chang Liu:** Formal analysis. **Liu He:** Validation, Data curation. **Jianqin Li:** Formal analysis. **Chao Sun:** Formal analysis. **Francis Martin:** Writing - review & editing. **Jingyuan Song:** Supervision, Methodology. **Shilin Chen:** Conceptualization, Funding acquisition. All authors read and approved the final manuscript.

## Competing interests

The authors have declared no competing interests.

## Supplementary material

Supplementary Figure S1 **Analysis of the genome sequencing depth**. *X*-axis represents sequencing depth, and *Y*-axis represents the ratio of the number of bases of a depth to the total number of bases. Using SOAPaligner to compare the short reads to the assembly genome sequence with less than 5 mismatches. The number of covered times for each base was counted according to the alignment results. Then the percentage of bases at various sequencing depths was obtained at the entire genome. The bases with the coverage depth lower than 10× were less than 2% among the genome scale.

Supplementary Figure S2 **Sequence slignment of *W. cocos* ITS2**. The ITS2 labeled JGI represented the ITS2 isolated from the American *W. cocos* strain. The ITS2 labeled IMPLAD was generated from the Chinese *W. cocos* strain CGMCC5.78 in this study. The other two ITS2 sequences (AY728272.1 and EF397597.1) were downloaded from GenBank.

Supplementary Figure S3 **Evolution analysis of *W. cocos***. **A.** Comparison of orthologous genes among the genomes of *W. cocos* (JGI), *W. cocos* (IMPLAD), *P. placenta*, *P. chrysosporium*, *L. Bicolor*, and *S. commune*. **B**. Venn diagram showing shared orthologous groups among the genomes of *W. cocos* (JGI), *W. cocos* (IMPLAD), *P. placenta*, *P. chrysosporium*, *L. Bicolor*, and *S. commune*. **C**. Phylogenetic tree and divergence time analysis of *W. cocos*. The blue numbers on the nodes were the divergence times. The phylogenetic tree of the genomes of *W.cocos* (JGI), *W. cocos* (IMPLAD), *P. placenta*, *P. chrysosporium*, *L. Bicolor*, and *S. commune* was constructed using single-copy orthologuous genes. The molecular clock for the first phase sites in species was estimated with single-copy orthologous genes. The divergence times were estimated among all species. **D**. Gene family expansion and contraction in *W.cocos* compared to *P. placenta*, *P. chrysosporium*, *L. Bicolor*, and *S. Commune* was identified by CAFE (version 2.1) with the default parameters based on the phylogenetic analysis. MYA, million years ago.

Supplementary Figure S4 **The mating genes in *W. cocos***. **A.** The HD loci located in the scaffold 4 in *W. cocos* (IMPLAD) genome. **B.** The MIP and β-FG loci located in the scaffold 1 in *W. cocos* (JGI) genome. **C.** The STE3-like pheromone receptors located in the scaffold 6 in W. cocos (JGI) genome.

Supplementary Figure S5 **The fruiting body of *W. cocos***. **A.** The fruiting body grown in a petri dish after the mycelia were cultured on PDA medium (Potato Dextrose Agar medium) for 3 months. **B.** The enlarged image of the *W. cocos* fruiting body.

Supplementary Figure S6 **The phylogeny of velvet proteins in *W. cocos***. These velvet proteins were identified from the genomes of *W. cocos*, *G. lucidum*, *A. nidulans*, and *U. maydis*. The WCO008289.1 and WCO005599.1 were predicted to be VosA and VelB, respectively.

Supplementary Figure S7 **The predicted mechanism of sclerotial formation in *W. cocos***. The signaling molecules, velvet proteins, transcription factors, transporters, and the genes related with secondary metabolism and wood degradation were predicted to be invovled in the formation processes of sclerotium of *W. cocos*.
