# Supplementary figures and images for: The Wolfiporia cocos Genome and Transcriptome Shed Light on the Formation of Its Edible and Medicinal Sclerotium
Source: Genomics Proteomics Bioinformatics. 2020 Dec 24;18(4):455–67. doi: 10.1016/j.gpb.2019.01.007 (PMC8242266; doi:10.1016/j.gpb.2019.01.007)

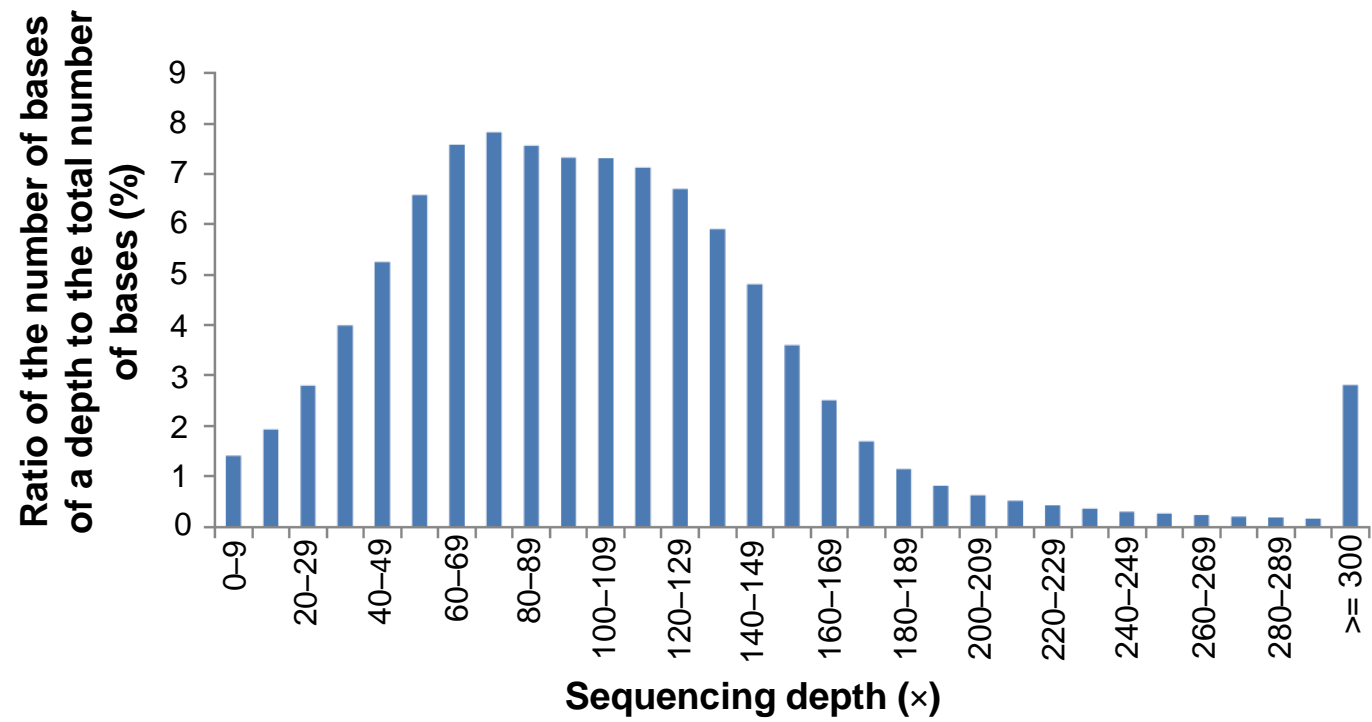

Supplement: Supplementary Figure S1 — Analysis of the genome sequencing depth. X-axis represents sequencing depth, and Y-axis represents the ratio of the number of bases of a depth to the total number of bases. Using SOAPaligner to compare the short reads to the assembly genome sequence with less than 5 mismatches. The number of covered times for each base was counted according to the alignment results. Then the percentage of bases at various sequencing depths was obtained at the entire genome. The bases with the coverage depth lower than 10× were less than 2% among the genome scale. [file mmc1.pdf]

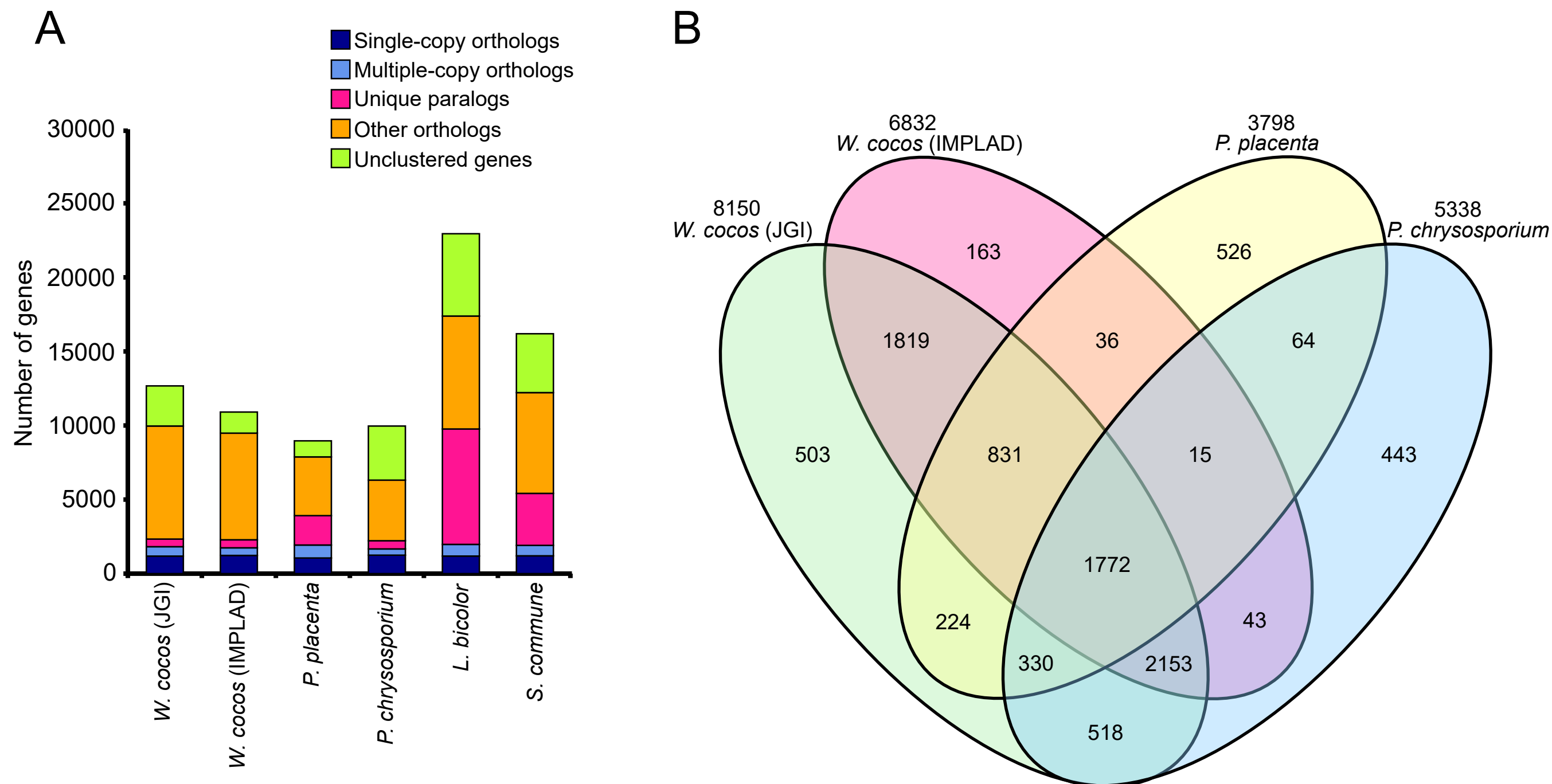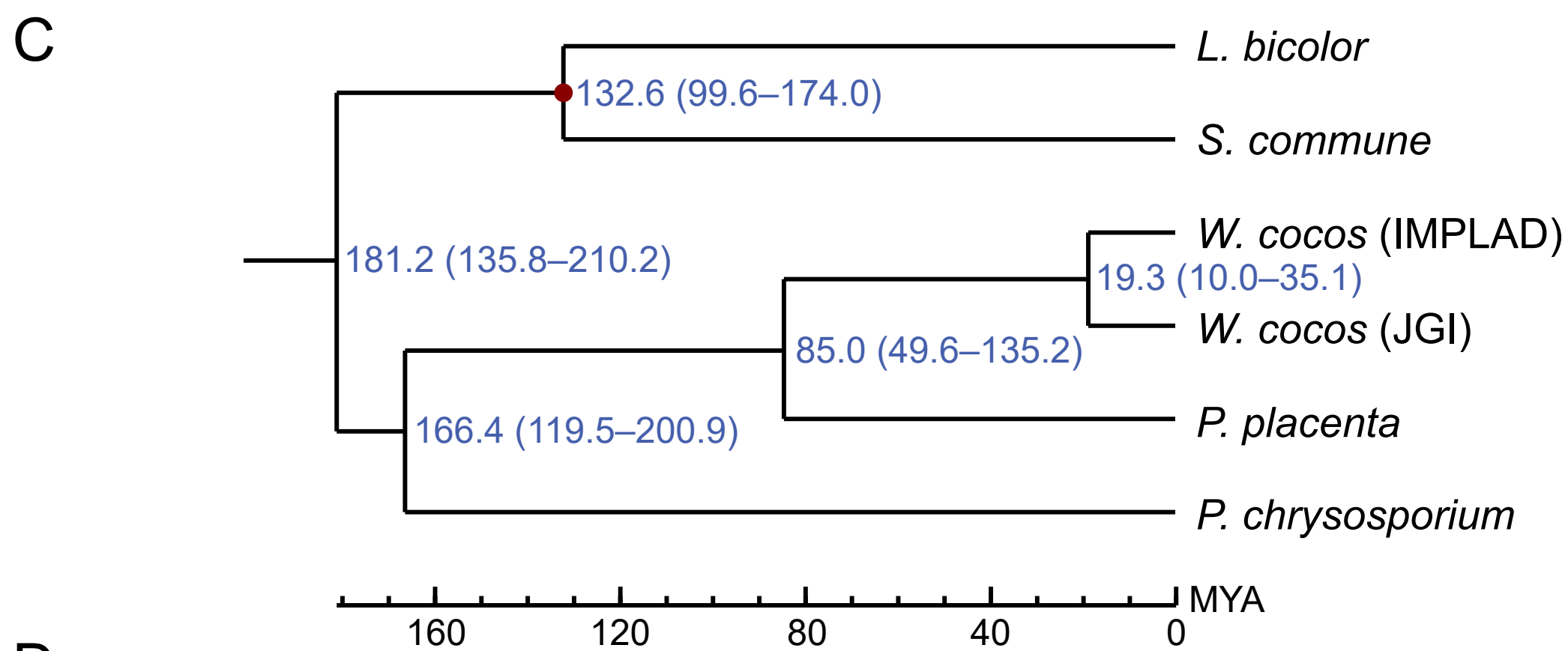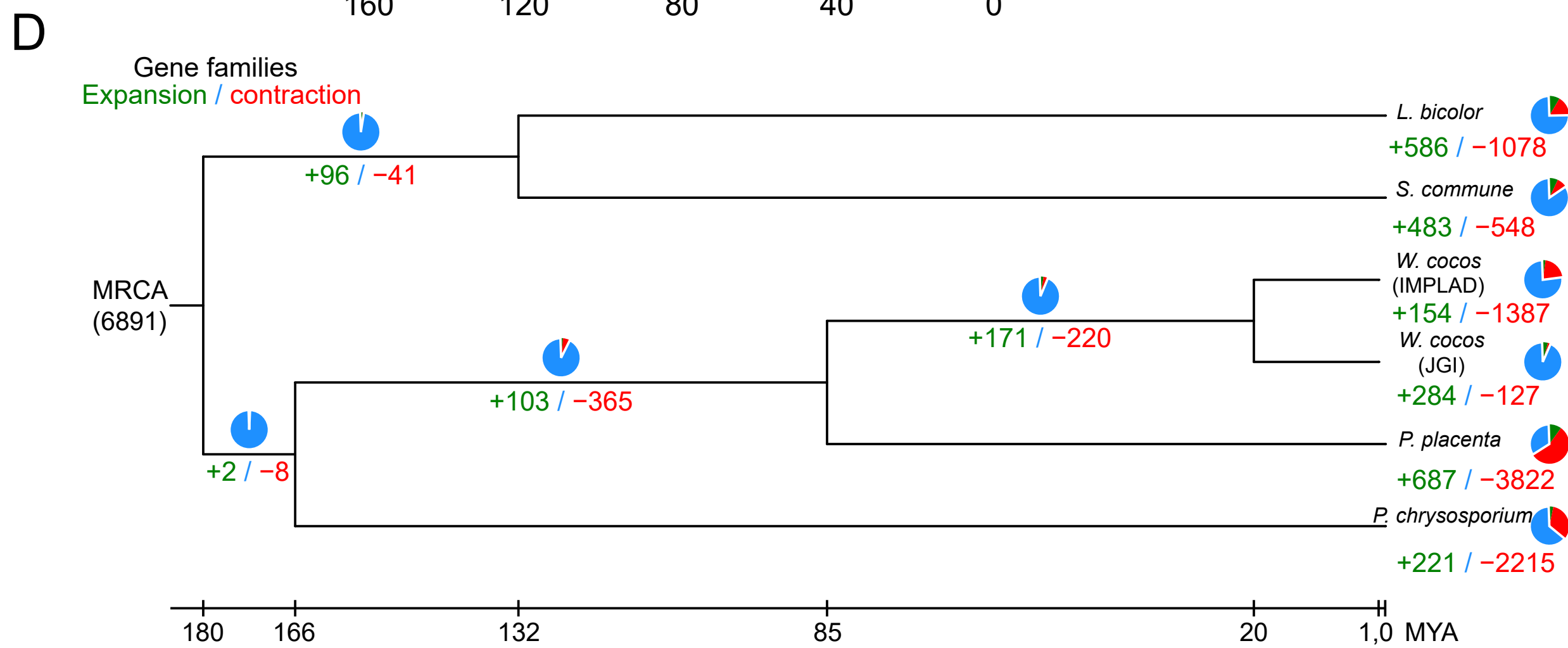

Supplement: Supplementary Figure S3 — Evolution analysis of W. cocos. A. Comparison of orthologous genes among the genomes of W. cocos (JGI), W. cocos (IMPLAD), P. placenta, P. chrysosporium, L. Bicolor, and S. commune. B. Venn diagram showing shared orthologous groups among the genomes of W. cocos (JGI), W. cocos (IMPLAD), P. placenta, P. chrysosporium, L. Bicolor, and S. commune. C. Phylogenetic tree and divergence time analysis of W. cocos. The blue numbers on the nodes were the divergence times. The phylogenetic tree of the genomes of W.cocos (JGI), W. cocos (IMPLAD), P. placenta, P. chrysosporium, L. Bicolor, and S. commune was constructed using single-copy orthologuous genes. The molecular clock for the first phase sites in species was estimated with single-copy orthologous genes. The divergence times were estimated among all species. D. Gene family expansion and contraction in W.cocos compared to P. placenta, P. chrysosporium, L. Bicolor, and S. Commune was identified by CAFE (version 2.1) with the default parameters based on the phylogenetic analysis. MYA, million years ago. [file mmc3.pdf]

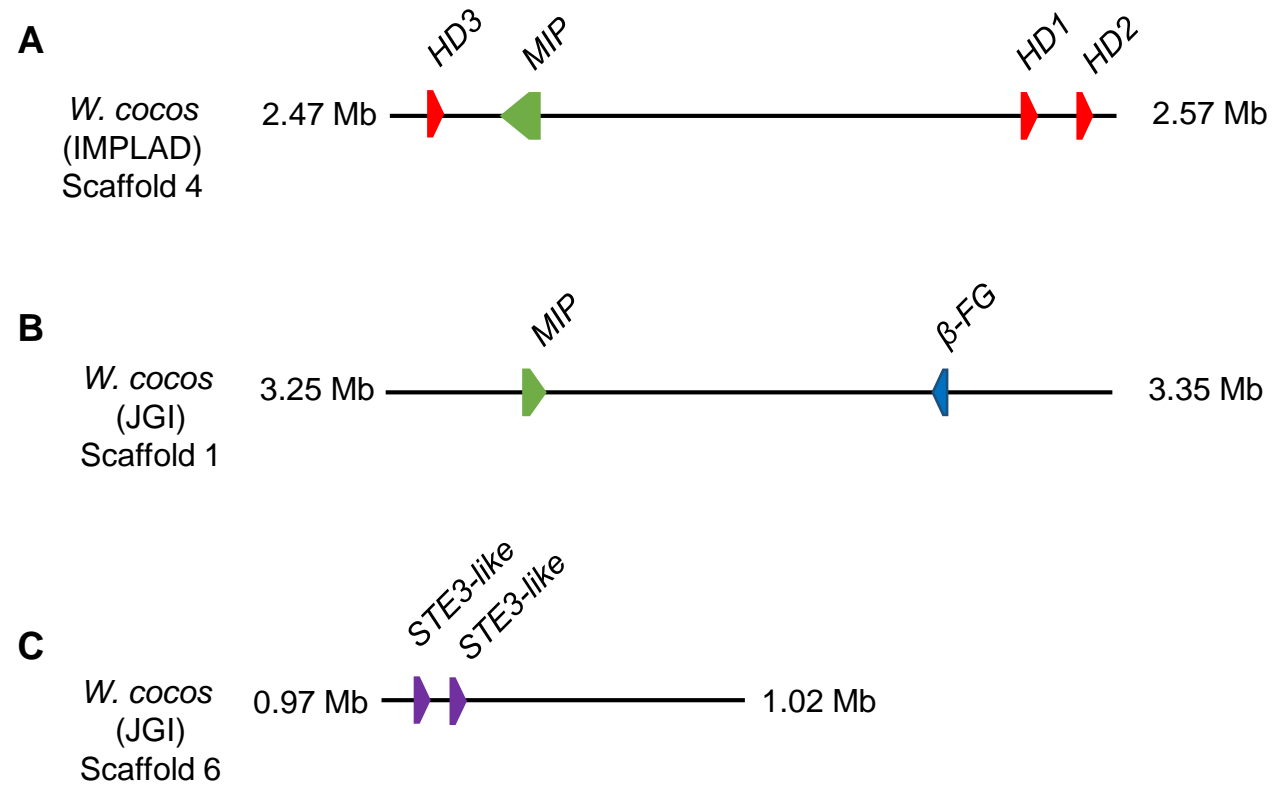

Supplement: Supplementary Figure S4 — The mating genes in W. cocos. A. The HD loci located in the scaffold 4 in W. cocos (IMPLAD) genome. B. The MIP and β-FG loci located in the scaffold 1 in W. cocos (JGI) genome. C. The STE3-like pheromone receptors located in the scaffold 6 in W. cocos (JGI) genome. [file mmc4.pdf]

## Slide 1
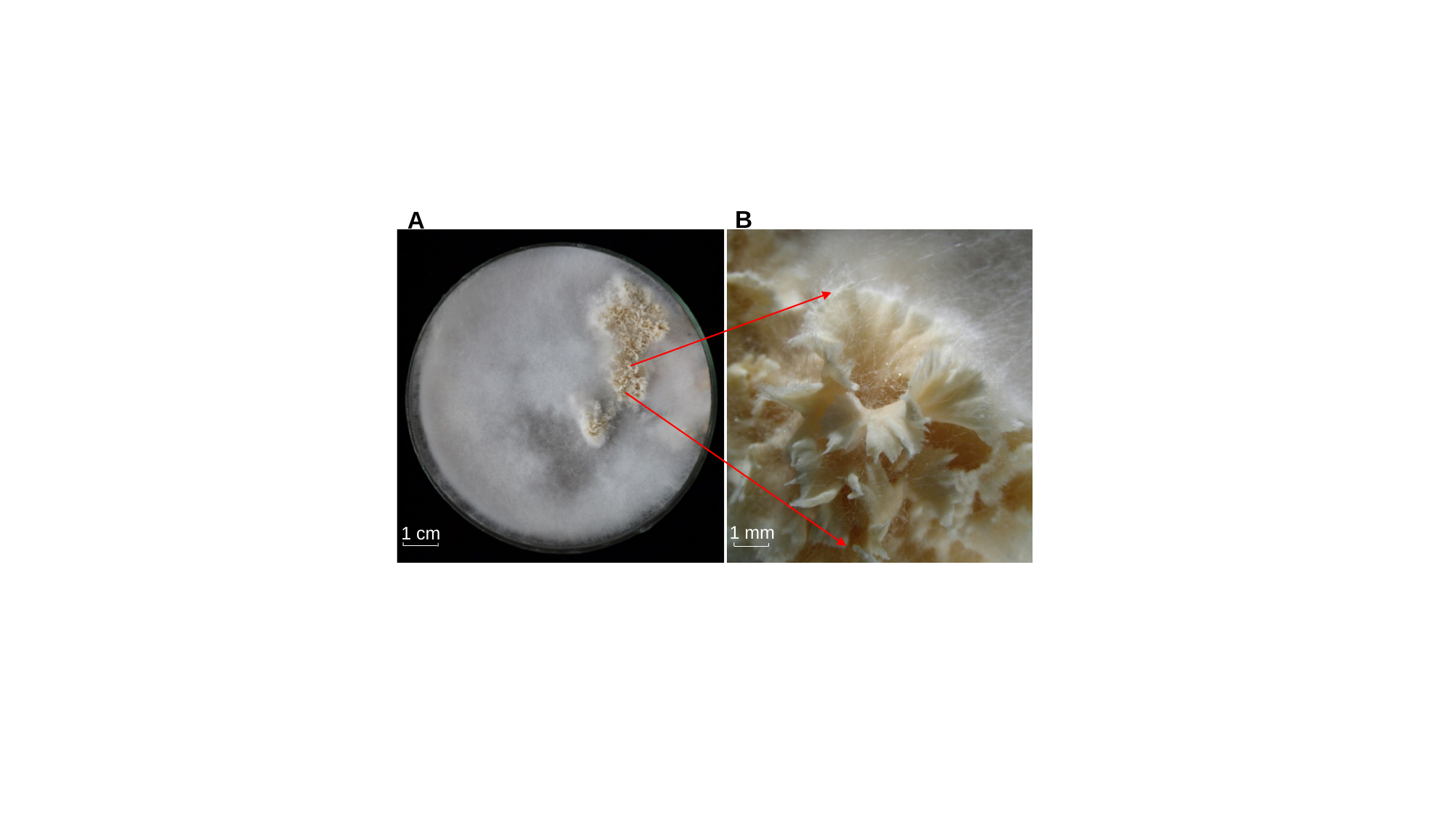

B
A
1 mm
1 cm

Supplement: Supplementary Figure S5 — The fruiting body of W. cocos. A. The fruiting body grown in a petri dish after the mycelia were cultured on PDA medium (Potato Dextrose Agar medium) for 3 months. B. The enlarged image of the W. cocos fruiting body. [file mmc5.pptx]

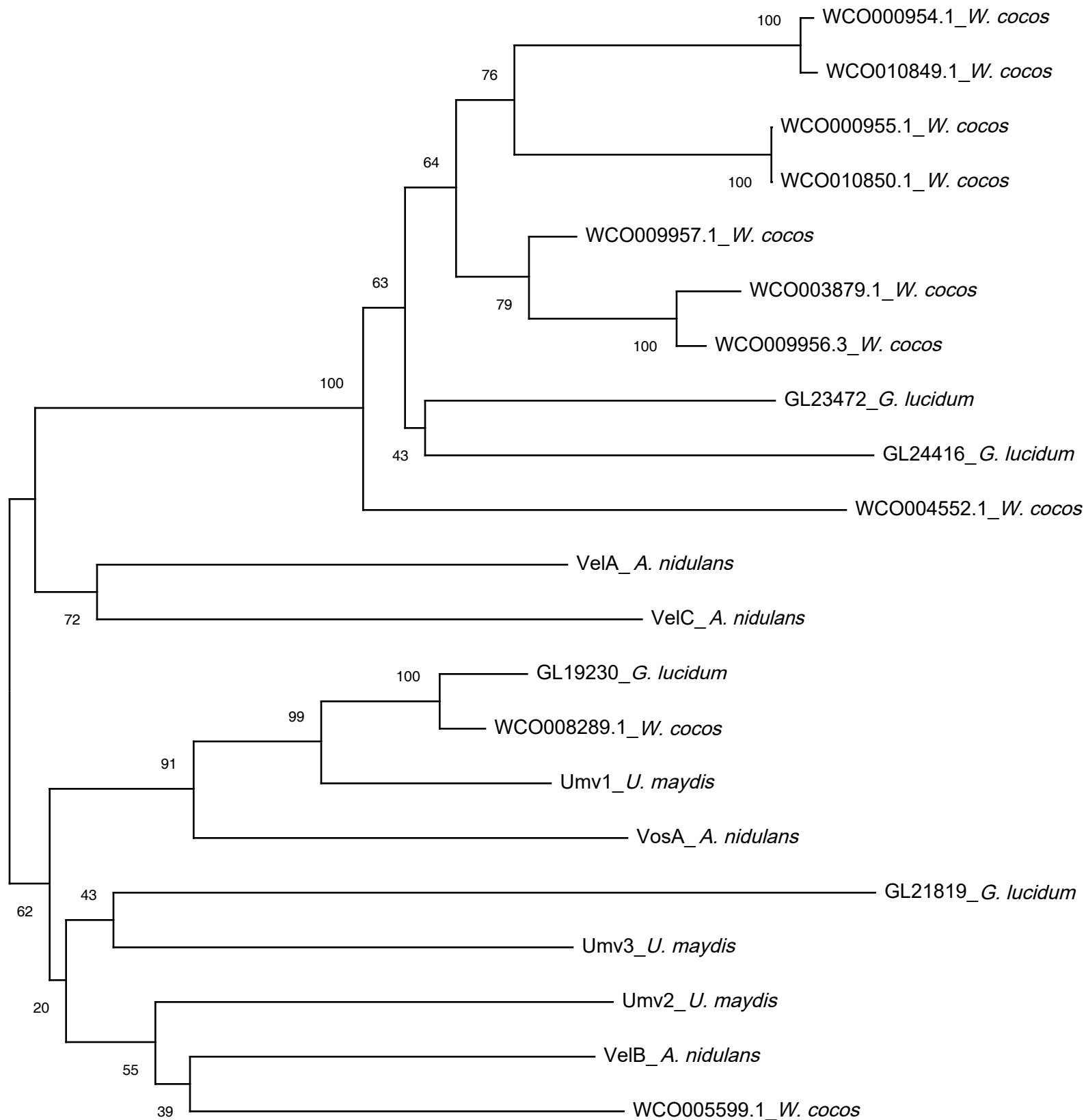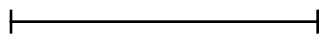

0.20

Supplement: Supplementary Figure S6 — The phylogeny of velvet proteins in W. cocos. These velvet proteins were identified from the genomes of W. cocos, G. lucidum, A. nidulans, and U. maydis. The WCO008289.1 and WCO005599.1 were predicted to be VosA and VelB, respectively. [file mmc6.pdf]

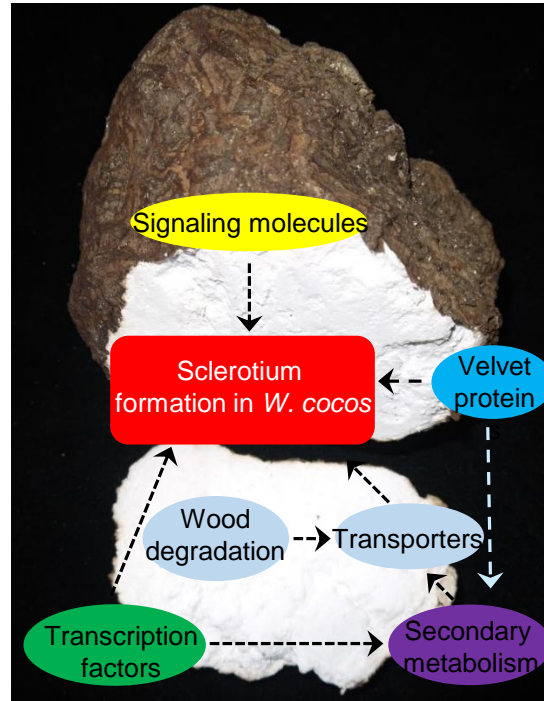

Supplement: Supplementary Figure S7 — The predicted mechanism of sclerotial formation in W. cocos. The signaling molecules, velvet proteins, transcription factors, transporters, and the genes related with secondary metabolism and wood degradation were predicted to be invovled in the formation processes of sclerotium of W. cocos. [file mmc7.pdf]
