# Supplementary material for: The Wolfiporia cocos Genome and Transcriptome Shed Light on the Formation of Its Edible and Medicinal Sclerotium
Source: Genomics Proteomics Bioinformatics. 2020 Dec 24;18(4):455–67. doi: 10.1016/j.gpb.2019.01.007 (PMC8242266; doi:10.1016/j.gpb.2019.01.007)
Supplement: Supplementary Figure S2 — Sequence slignment of W. cocos ITS2. The ITS2 labeled JGI represented the ITS2 isolated from the American W. cocos strain. The ITS2 labeled IMPLAD was generated from the Chinese W. cocos strain CGMCC5.78 in this study. The other two ITS2 sequences (AY728272.1 and EF397597.1) were downloaded from GenBank. [file mmc2.pdf]

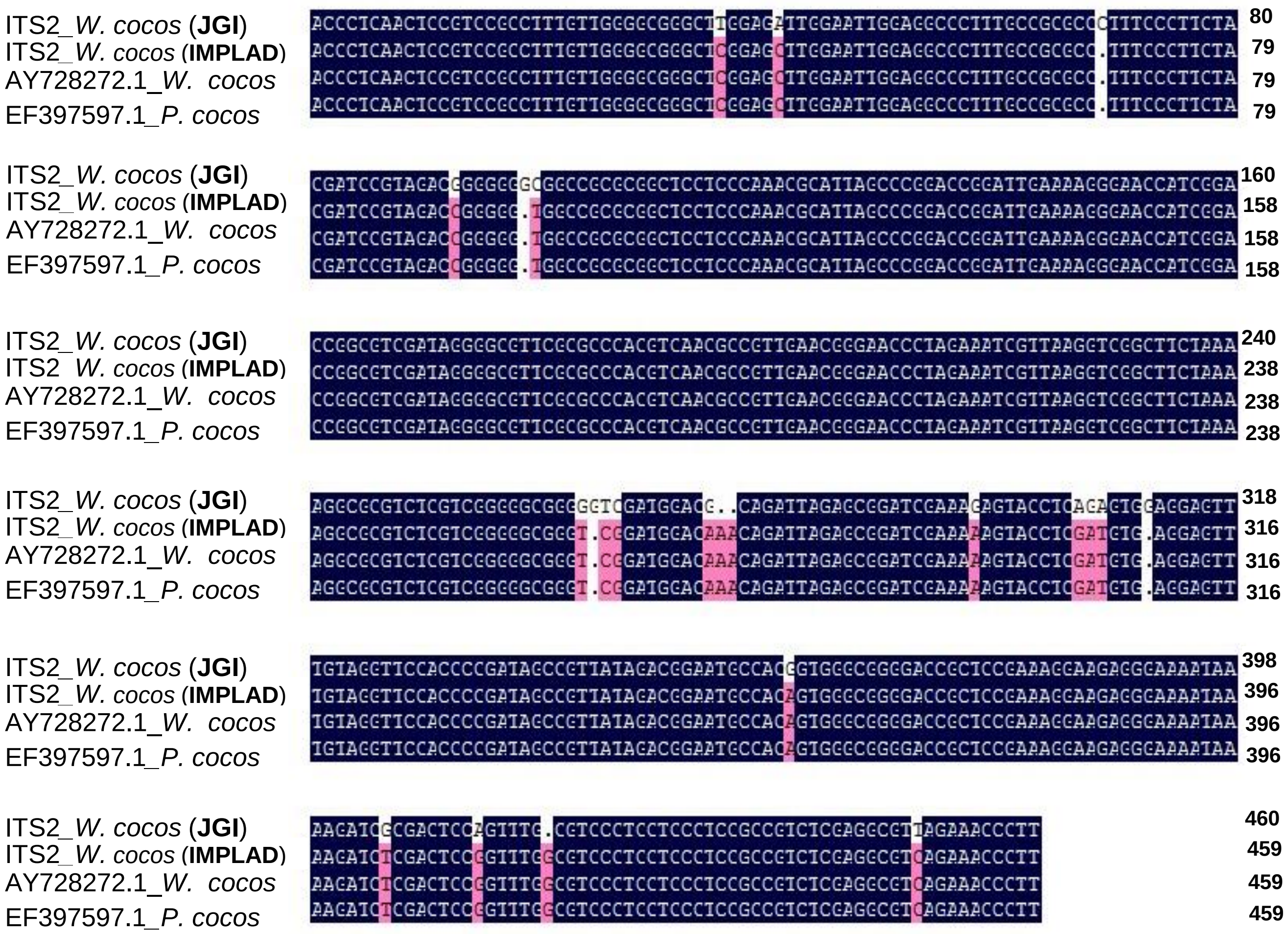
ITS2_W. cocos (JGI)
ITS2_W. cocos (IMPLAD)
AY728272.1_W. cocos
EF397597.1_P. cocos
80
79
79
79
160
158
158
158
240
238
238
238
318
316
316
316
398
396
396
396
460
459
459
459
