# Supplementary material for: The Wolfiporia cocos Genome and Transcriptome Shed Light on the Formation of Its Edible and Medicinal Sclerotium
Source: Genomics Proteomics Bioinformatics. 2020 Dec 24;18(4):455–67. doi: 10.1016/j.gpb.2019.01.007 (PMC8242266; doi:10.1016/j.gpb.2019.01.007)
Supplement: Supplementary data 9 [file mmc9.docx]

**Table S2 Summary of *W. cocos* genome sequencing data**

| Sequencing method | | Paired-end  insert size (bp) | Total data (Gb) | Read length (bp) | Sequence  coverage (×) |
| --- | --- | --- | --- | --- | --- |
| Solexa Reads | WGS | 511 | 7.90 | 100 | 158 |
|  |  | 2000 | 1.83 | 70 | 37 |
|  |  | 5000 | 2.14 | 70 | 43 |
|  |  | 10,000 | 2.60 | 49 | 52 |
|  |  | 20,000 | 0.81 | 49 | 16 |
|  |  | Subtotal | 15.28 |  | 306 |
|  | Fosmid | 250 | 66.92 | 88 | 1338 |
|  |  | 500 | 54.75 | 88 | 1095 |
|  |  | Subtotal | 121.68 |  | 2434 |
| Total |  |  | 136.95 |  | 2739 |

*Note*: The predicted genome size is 50.6 Mb. WGS, whole genome sequencing.
