# Supplementary material for: The Wolfiporia cocos Genome and Transcriptome Shed Light on the Formation of Its Edible and Medicinal Sclerotium
Source: Genomics Proteomics Bioinformatics. 2020 Dec 24;18(4):455–67. doi: 10.1016/j.gpb.2019.01.007 (PMC8242266; doi:10.1016/j.gpb.2019.01.007)
Supplement: Supplementary data 10 [file mmc10.docx]

**Table S3 Genome assembly of *W. cocos***

|  | **Contig** | |  | **Scaffold** | |
| --- | --- | --- | --- | --- | --- |
|  | **Size (bp)** | **Number** |  | **Size (bp)** | **Number** |
| N90 | 20,789 | 585 |  | 180,821 | 70 |
| N80 | 37,943 | 418 |  | 270,053 | 48 |
| N70 | 54,854 | 311 |  | 421,610 | 33 |
| N60 | 70,385 | 233 |  | 638,197 | 23 |
| N50 | 86,253 | 171 |  | 834,581 | 16 |
| Longest | 371,550 | - |  | 2,999,645 | - |
| Total size | 48,858,798 | - |  | 50,617,620 | - |
| Total number (>100 bp) | - | 1433 |  | - | 351 |
| Total number (>2 kb) | - | 1124 |  | - | 249 |
