# Supplementary material for: The Wolfiporia cocos Genome and Transcriptome Shed Light on the Formation of Its Edible and Medicinal Sclerotium
Source: Genomics Proteomics Bioinformatics. 2020 Dec 24;18(4):455–67. doi: 10.1016/j.gpb.2019.01.007 (PMC8242266; doi:10.1016/j.gpb.2019.01.007)
Supplement: Supplementary data 11 [file mmc11.docx]

**Table S4 Comparison of the assembled *W. cocos* whole genome with the sequences of five fosmid clones**

| Fosmid clone_ID | Length (bp) | Coverage ratio (%) | Alignment block number | Assembly block number | Scaffold number | Scaffold length (bp) | Gap number | Gap length (bp) |
| --- | --- | --- | --- | --- | --- | --- | --- | --- |
| yzqaxa | 21,885 | 100 | 1 | 1 | 7 | 2,907,922 | 0 | 0 |
| yzqbxa | 35,223 | 99.2249 | 3 | 3 | 8 | 1,195,326 | 1 | 470 |
| yzqcxa | 26,349 | 99.9962 | 1 | 1 | 32 | 656,467 | 0 | 0 |
| yzqdxa | 29,925 | 96.5914 | 3 | 3 | 31 | 417,174 | 1 | 816 |
| yzqexa | 23,672 | 100 | 1 | 1 | 8 | 212,162 | 0 | 0 |
