# Supplementary material for: The Wolfiporia cocos Genome and Transcriptome Shed Light on the Formation of Its Edible and Medicinal Sclerotium
Source: Genomics Proteomics Bioinformatics. 2020 Dec 24;18(4):455–67. doi: 10.1016/j.gpb.2019.01.007 (PMC8242266; doi:10.1016/j.gpb.2019.01.007)
Supplement: Supplementary data 12 [file mmc12.docx]

**Table S5 Gene prediction of the *W. cocos* genome**

| **Gene set** | | **No. of genes** | **Average gene length (bp)** | **Average CDS length (bp)** | **Average exon per gene** | **Average exon length (bp)** | **Average intron length (bp)** |
| --- | --- | --- | --- | --- | --- | --- | --- |
| *De novo* | Augustus | 9708 | 1679.72 | 1356.78 | 5.21 | 260.49 | 76.74 |
|  | Genemark_ES | 12,432 | 1982.04 | 1449.70 | 5.80 | 249.88 | 110.87 |
|  | SNAP | 12,877 | 1403.84 | 1160.30 | 4.16 | 278.61 | 76.96 |
| Homolog | *P. chrysoporium* | 8737 | 1455.45 | 1084.69 | 4.77 | 227.63 | 98.47 |
|  | *P. placenta* | 9325 | 1669.74 | 1167.17 | 5.22 | 223.55 | 119.06 |
|  | Swissprot | 5876 | 1048.33 | 797.03 | 3.77 | 211.63 | 90.85 |
| GLEAN |  | 11,935 | 1789.06 | 1356.41 | 5.18 | 261.80 | 103.48 |
| Final (combined with RNA-Seq) | | 10,908 | 1829.00 | 1365.99 | 5.09 | 268.49 | 91.84 |

*Note*: All the transcripts in this table did not include the UTR regions. The database of Swissprot (release-2011-08) was used to annotate the homological proteins.
