# Supplementary material for: The Wolfiporia cocos Genome and Transcriptome Shed Light on the Formation of Its Edible and Medicinal Sclerotium
Source: Genomics Proteomics Bioinformatics. 2020 Dec 24;18(4):455–67. doi: 10.1016/j.gpb.2019.01.007 (PMC8242266; doi:10.1016/j.gpb.2019.01.007)
Supplement: Supplementary data 13 [file mmc13.docx]

**Table S6 Gene annotation of *W. cocos* genes**

|  | Public database | No. of genes | Percent (%) |
| --- | --- | --- | --- |
| No. of total genes |  | 10,908 | - |
| No. of annotated genes |  | 9277 | 85.05 |
| Annotated in Databases | InterPro | 6405 | 58.72 |
|  | GO | 5263 | 48.25 |
|  | KEGG | 5413 | 49.62 |
|  | Swissprot | 6070 | 55.65 |
|  | TrEMBL | 9116 | 83.57 |
|  | nt | 9113 | 83.54 |
|  | nr | 8648 | 79.28 |
|  | KOG | 5275 | 48.36 |
|  | COG | 3597 | 32.98 |
| Unannotated |  | 1631 | 14.95 |

*Note*: GO, Gene Ontology; KEGG, Kyoto Encyclopedia of Genes and Genomes; KOG, Clusters of orthologous groups for eukaryotic complete genomes; COG, Clusters of Orthologous Groups of proteins.
