# Supplementary material for: The Wolfiporia cocos Genome and Transcriptome Shed Light on the Formation of Its Edible and Medicinal Sclerotium
Source: Genomics Proteomics Bioinformatics. 2020 Dec 24;18(4):455–67. doi: 10.1016/j.gpb.2019.01.007 (PMC8242266; doi:10.1016/j.gpb.2019.01.007)
Supplement: Supplementary data 14 [file mmc14.docx]

**Table S7 RNA annotation in the *W. cocos* genome**

| **Type** | | **No. of genes** | **Average length (bp)** | **Total length (bp)** | **Percentage of genome (%)** |
| --- | --- | --- | --- | --- | --- |
| tRNA |  | 184 (including 29 pseudogenes ) | 84.92 | 13,163 | 0.02600 |
| rRNA | rRNA | 7 | 237.43 | 1662 | 0.00328 |
|  | 18S | 5 | 271.40 | 1357 | 0.00268 |
|  | 26S | 1 | 163 | 163 | 0.00032 |
|  | 5.8S | 1 | 142 | 142 | 0.00028 |
| snRNA | snRNA | 15 | 135.73 | 2036 | 0.00402 |
|  | CD-box | 4 | 98.75 | 395 | 0.00078 |
|  | Splicing | 11 | 149.18 | 1641 | 0.00324 |
