# Supplementary material for: The Wolfiporia cocos Genome and Transcriptome Shed Light on the Formation of Its Edible and Medicinal Sclerotium
Source: Genomics Proteomics Bioinformatics. 2020 Dec 24;18(4):455–67. doi: 10.1016/j.gpb.2019.01.007 (PMC8242266; doi:10.1016/j.gpb.2019.01.007)
Supplement: Supplementary data 15 [file mmc15.docx]

**Table S8 Proportions of single-copy orthologs, multiple-copy orthologs, unique paralogs, other orthologs, and unclustered genes in the fungi genomes.**

| **Species** | **Proportion of single-copy orthologs (%)** | **Proportion of multiple-copy orthologs (%)** | **Proportion of unique paralogs (%)** | **Proportion of other orthologs (%)** | **Proportion of unclustered genes (%)** |
| --- | --- | --- | --- | --- | --- |
| *A. nidulans* | 5.68 | 1.37 | 5.67 | 63.15 | 24.13 |
| *C. cinerea* | 4.52 | 1.34 | 12.44 | 62.12 | 19.56 |
| *C. gattii* | 9.23 | 0.47 | 0.53 | 83.12 | 6.64 |
| *C. neoformans* | 9.21 | 0.49 | 0.58 | 86.49 | 3.24 |
| *L. bicolor* | 2.64 | 1.09 | 31.43 | 46.69 | 18.15 |
| *M. laricis* | 3.71 | 1.07 | 35.97 | 41.85 | 17.41 |
| *N. crassa* | 6.12 | 0.48 | 4.59 | 61.11 | 27.70 |
| *P. chrysosporium* | 6.18 | 1.86 | 9.27 | 69.76 | 12.92 |
| *P. placenta* | 6.06 | 4.28 | 20.23 | 57.77 | 11.66 |
| *P. graminis* | 2.95 | 1.23 | 36.60 | 38.89 | 20.33 |
| *S. cerevisiae* | 9.22 | 3.77 | 6.92 | 49.46 | 30.63 |
| *S. commune* | 4.16 | 1.59 | 16.63 | 59.55 | 18.07 |
| *S. lacrymans* | 4.30 | 1.10 | 18.08 | 55.24 | 21.28 |
| *U. maydis* | 9.29 | 0.66 | 3.53 | 66.64 | 19.89 |
| *W. cocos* | 5.56 | 1.72 | 11.67 | 67.85 | 13.20 |
