# Supplementary material for: The Wolfiporia cocos Genome and Transcriptome Shed Light on the Formation of Its Edible and Medicinal Sclerotium
Source: Genomics Proteomics Bioinformatics. 2020 Dec 24;18(4):455–67. doi: 10.1016/j.gpb.2019.01.007 (PMC8242266; doi:10.1016/j.gpb.2019.01.007)
Supplement: Supplementary data 17 [file mmc17.docx]

**Table S10 Conservation of *W. cocos* genes in OrthoMCL families**

|  | **Gene family** | | **Gene** | |
| --- | --- | --- | --- | --- |
|  | ***W. cocos* (IMPLAD)** | ***W. cocos***  **(JGI)** | ***W. cocos***  **(IMPLAD)** | ***W. cocos***  **(JGI)** |
| No. of single-copy genes | - | - | 1081 | 1081 |
| No. of paralogs | 741 | 741 | 1047 | 1087 |
| No. of genes specific to Polyrales | 114 | 114 | 161 | 175 |
| No. of genes specific to *Wolfiporia* | 694 | 1120 | 694 | 950 |
| No. of strain-specific genes | 120 | 148 | 455 | 432 |
| Total | 6727 | 8245 | 10,908 | 12,746 |
