# Supplementary material for: The Wolfiporia cocos Genome and Transcriptome Shed Light on the Formation of Its Edible and Medicinal Sclerotium
Source: Genomics Proteomics Bioinformatics. 2020 Dec 24;18(4):455–67. doi: 10.1016/j.gpb.2019.01.007 (PMC8242266; doi:10.1016/j.gpb.2019.01.007)
Supplement: Supplementary data 18 [file mmc18.docx]

**Table S11 The whole-genome comparison of the *W. cocos* strain (IMPLAD) against the American *W. cocos* strain (JGI) using Mummer**

| Sequence identity (%) | Total length of sequences (bp) | Percentage of reads in the total reads (%) |
| --- | --- | --- |
| ≥70 | 31,824,694 | 62.87 |
| ≥80 | 31,791,576 | 62.80 |
| ≥90 | 29,821,820 | 58.92 |
| ≥95 | 22,723,802 | 44.89 |
