# Supplementary material for: The Wolfiporia cocos Genome and Transcriptome Shed Light on the Formation of Its Edible and Medicinal Sclerotium
Source: Genomics Proteomics Bioinformatics. 2020 Dec 24;18(4):455–67. doi: 10.1016/j.gpb.2019.01.007 (PMC8242266; doi:10.1016/j.gpb.2019.01.007)
Supplement: Supplementary data 19 [file mmc19.docx]

**Table S12 Matching of *W. cocos* mycelium and sclerotium transcriptome data to the genome**

|  | **Mycelium** | | **Sclerotium** | |
| --- | --- | --- | --- | --- |
|  | **Reads number** | **Percentage** | **Reads number** | **Percentage** |
| Total reads | 51,222,268 |  | 55,340,386 |  |
| Total base pairs | 4,610,004,120 |  | 4,980,634,740 |  |
| Total mapped reads | 37,499,521 | 73.21% | 35,726,989 | 64.56% |
| Perfect match | 28,454,261 | 55.55% | 27,026,805 | 48.84% |
| <=3 bp mismatch | 9,045,260 | 17.66% | 8,700,184 | 15.72% |
| unique match | 36,751,775 | 71.75% | 34,934,440 | 63.13% |
| multi-position match | 747,746 | 1.46% | 792,549 | 1.43% |
| Total unmapped reads | 13,722,747 | 26.79% | 19,613,397 | 35.44% |
