# Supplementary material for: The Wolfiporia cocos Genome and Transcriptome Shed Light on the Formation of Its Edible and Medicinal Sclerotium
Source: Genomics Proteomics Bioinformatics. 2020 Dec 24;18(4):455–67. doi: 10.1016/j.gpb.2019.01.007 (PMC8242266; doi:10.1016/j.gpb.2019.01.007)
Supplement: Supplementary data 20 [file mmc20.docx]

**Table S13 Overview of the transcriptome datasets of *W. cocos* mycelium and sclerotium**

|  | **Mycelium** | **Sclerotium** |
| --- | --- | --- |
| No. of genes | 8395 | 8479 |
| Average length of genes (bp) | 1417 | 1408 |
| No. of contigs | 8298 | 8398 |
| Average length of contigs (bp) | 1426 | 1417 |
| No. of singletons | 97 | 81 |
| Average length of singletons (bp) | 615 | 483 |
| No. of total genes | 8548 | |
