# Supplementary material for: The Wolfiporia cocos Genome and Transcriptome Shed Light on the Formation of Its Edible and Medicinal Sclerotium
Source: Genomics Proteomics Bioinformatics. 2020 Dec 24;18(4):455–67. doi: 10.1016/j.gpb.2019.01.007 (PMC8242266; doi:10.1016/j.gpb.2019.01.007)
Supplement: Supplementary data 21 [file mmc21.docx]

**Table S14 Distribution of gene coverage in the transcriptomes of *W. cocos* mycelium and sclerotium**

| **Gene coverage** | **No. of genes in mycelium** | **No. of genes in sclerotium** |
| --- | --- | --- |
| 90%–100% | 6511 | 6737 |
| 80%–90% | 659 | 617 |
| 70%–80% | 334 | 304 |
| 60%–70% | 221 | 220 |
| 50%–60% | 195 | 170 |
| 40%–50% | 140 | 115 |
| 30%–40% | 98 | 103 |
| 20%–30% | 103 | 102 |
| 10%–20% | 96 | 82 |
| 0%–10% | 38 | 29 |
