# Supplementary material for: The Wolfiporia cocos Genome and Transcriptome Shed Light on the Formation of Its Edible and Medicinal Sclerotium
Source: Genomics Proteomics Bioinformatics. 2020 Dec 24;18(4):455–67. doi: 10.1016/j.gpb.2019.01.007 (PMC8242266; doi:10.1016/j.gpb.2019.01.007)
Supplement: Supplementary data 31 [file mmc31.docx]

**Table S24 Loci of mating genes in the genomes of** ***W. cocos* (IMPLAD) and *W. cocos* (JGI)**

| **Mating gene** | ***W. cocos* (IMPLAD)** | | | ***W. cocos* (JGI)** | | |
| --- | --- | --- | --- | --- | --- | --- |
|  | **Gene ID** | **Scaffold** | **Location (bp)** | **Gene ID** | **Scaffold** | **Location**  **(bp)** |
| *HD1* | WCO006754.1 | Scaffold 4 | 2,562,309-2,563,302 | N/A | N/A | N/A |
| *HD2* | WCO006755.1 | Scaffold 4 | 2,563,764-2,564,971 | N/A | N/A | N/A |
| *HD3* | WCO006732.1 | Scaffold 4 | 2,484,006-2,485,892 | N/A | N/A | N/A |
| *MIP* | WCO006733.1 | Scaffold 4 | 2,489,246-2,494,401 | Wolco1\|112898 | Scaffold 1 | 3,287,430-3,290,347 |
| *beta-FG* | WCO000491.1 | Scaffold 1 | 2,146,223-2,147,721 | Wolco1\|15020 | Scaffold 1 | 3,322,807-3,324,529 |
| *STE3-like* | WCO006868.1 | Scaffold 40 | 185,249-186,735 | Wolco1\|141188 | Scaffold 24 | 187,349-189,318 |
| *STE3-like* | WCO007080.1 | Scaffold 42 | 199,526-200,827 | Wolco1\|136529 | Scaffold 3 | 673,452-675,463 |
| *STE3-like* | N/A | N/A | N/A | Wolco1\|25621 | Scaffold 6 | 987,281-989,308 |
| *STE3-like* | N/A | N/A | N/A | Wolco1\|73615 | Scaffold 6 | 974,599-975,220 |

*Note*: N/A represents the gene which was not identified in the genome.
